# Ypsilandrosides U-Y, five new steroidal saponins from *Ypsilandrathibetica*

**DOI:** 10.1007/s13659-022-00337-0

**Published:** 2022-05-05

**Authors:** Wen-Tao Gao, Ling-Ling Yu, Jing Xie, Long-Gao Xiao, Shi-Juan Zhang, Wen-Yi Ma, Huan Yan, Hai-Yang Liu

**Affiliations:** 1 College of Traditional Chinese Medicine, Yunnan University of Chinese Medicine, Kunming, 650500 China; 2 State Key Laboratory of Phytochemistry and Plant Resources in West China, and Yunnan Key Laboratory of Natural Medicinal Chemistry, Kunming Institute of Botany, Chinese Academy of Sciences, Kunming, 650201 China; 3 University of Chinese Academy of Sciences, Beijing, 100049 China

**Keywords:** *Ypsilandrathibetica*, Melanthiaceae, Ypsilandrosides U-Y, Spirostanol saponins, Cholestanol saponins

## Abstract

Phytochemical reinvestigation on the whole plants of *Ypsilandra* *thibetica* obtained four new spirostanol glycosides, named ypsilandrosides U-X (**1**–**4**), and one new cholestanol glycoside, named ypsilandroside Y (**5**). Their structures have been established by extensive spectroscopic data and chemical methods. Among them, compound **4** is a rare spirostanol glycoside which possesses a novel 5(6 → 7) abeo-steroidal aglycone, while compound **1** is a first spirostanol bisdesmoside attached to C-3 and C-12, respectively, isolated from the genus *Ypsilandra*. The induced platelet aggregation activity of the isolates was tested.

**Graphical Abstract:**

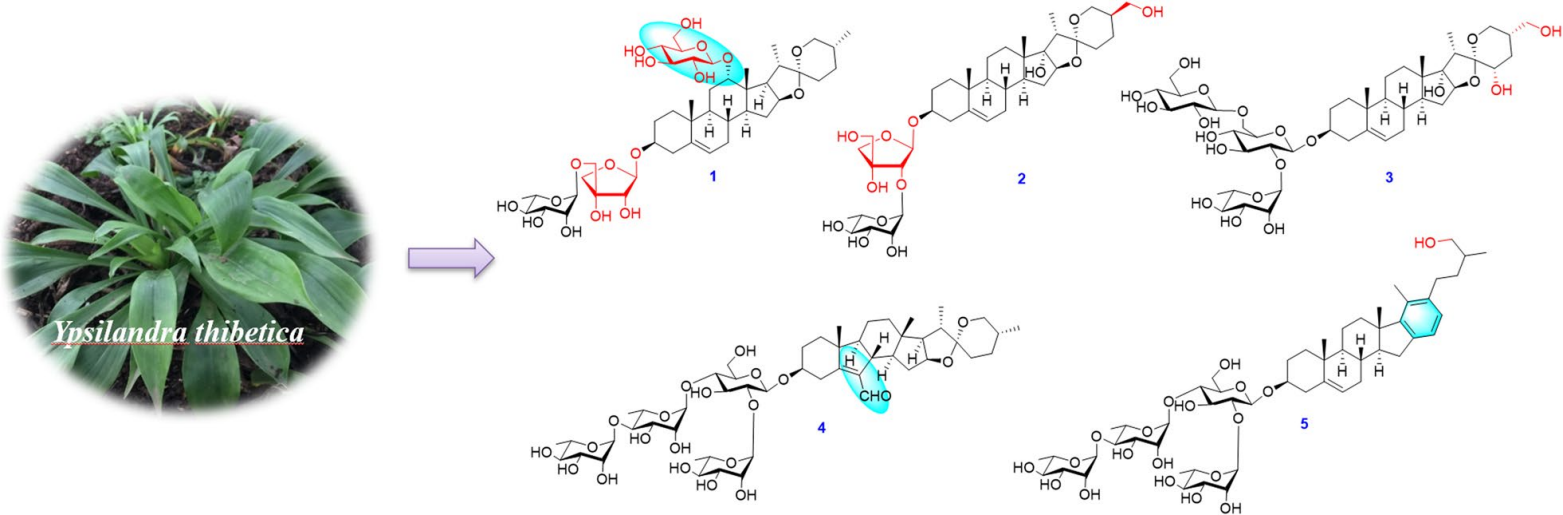

**Supplementary Information:**

The online version contains supplementary material available at 10.1007/s13659-022-00337-0.

## Introduction

**Fig. 1:**
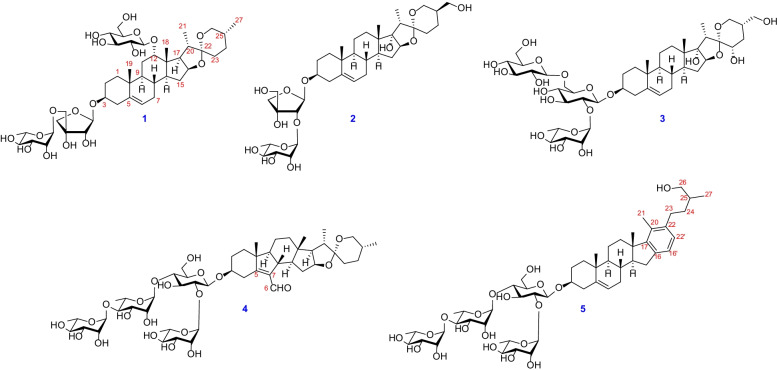
Chemical structures of saponins **1‒5**

*Ypsilandra* (Melanthiaceae) is distributed in southwestern China and Myanmar, which contains 5 species according to the updated classification of the Angiosperm Phylogeny Group IV [1]. Among them, *Ypsilandra* *thibetica* has been used in folk medicine for treatment of scrofula, dysuria, edema, uterine bleeding, and traumatic hemorrhage in China by the local people [2, 3]. Our previous investigations discovered twenty eight new steroidal glycosides including nineteen spirostanol saponins, two furostanol saponins, three cholestanol saponins, two pregnane glycosides, and two C_22_-steroidal lactone glycosides from this species [4–10], some of which showed cytotoxicity [4, 5], antifungal [4, 6], antibacterial [6], anti-HIV-1 activities [7], and so on. For further investigation on the chemical constituents of this herb, four new spirostanol saponins (**1**‒**4**) and one new cholestanol saponin (**5**) (Fig. 1) were obtained and structurally characterized. The current paper reports the isolation, structural elucidation, and the induced platelet aggregation activity of these isolates.

## Results and discussion

**Fig. 2:**
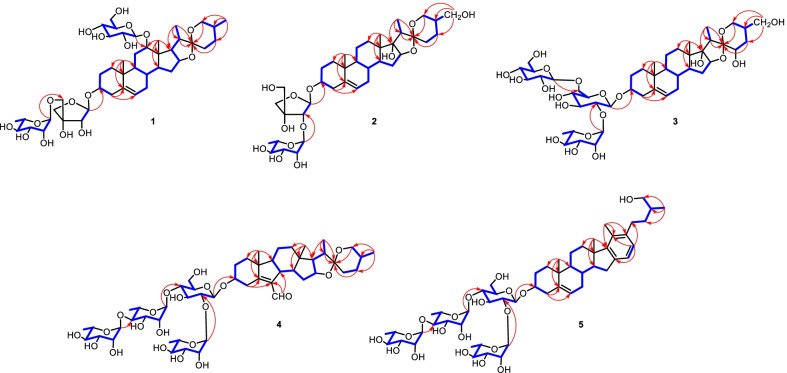
^1^H‒^1^H COSY and Key HMBC correlations of **1‒5**

**Fig. 3:**
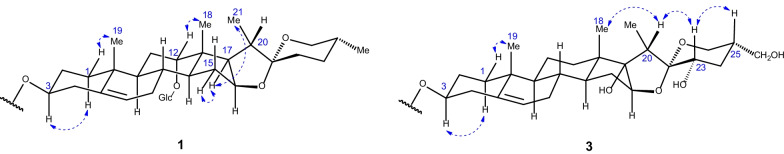
Key ROESY correlations for the aglycone moieties of **1** and **3**

Compound **1** was isolated as an amorphous powder. Its molecular formula was determined as C_44_H_70_O_17_ by the positive-ion HRESI-MS at *m/z* 893.4500 [M + Na]^+^ (calcd. for C_44_H_70_O_17_Na, 893.4505) and ^13^C NMR data (Table 2). The ^1^H NMR spectrum of **1** (Table 1) showed four methyl proton signals at *δ*_H_ 0.89 (s, CH_3_-18), 0.91 (s, CH_3_-19), 1.38 (d, *J* = 7.0 Hz, CH_3_-21), and 0.67 (d, *J* = 5.4 Hz, CH_3_-27), one olefinic proton signal at *δ*_H_ 5.18 (o, H-6), while three anomeric protons at *δ*_H_ 5.64 (d, *J* = 3.2 Hz, H-1*'*), 5.30 (br s, H-1*''*), and 4.86 (d, *J* = 7.8 Hz, H-1*'''*), which suggested that **1** was a glycoside with three monosaccharide moieties. The ^13^C NMR spectra displayed 44 carbon signals, of which 17 were assigned to those of one pentose and two hexose units, whereas other 27 ones were assigned to the aglycone moiety, including four methyl groups, nine methylene groups (one oxygenated), ten methine groups (one olefinic and three oxygenated), and four quaternary carbons (one olefinic and one ketal). The above NMR data suggested that compound **1** is a typical C-27 steroidal saponin and its aglycone is heloniogen [11]. This deduction can be confirmed by 2D-NMR spectra. The ^1^H‒^1^H COSY correlations revealed that the aglycone for **1** had four structural fragments as shown in (Fig. 2). Furthermore, the key HMBC correlations (Fig. 2) from CH_3_-18 (*δ*_H_ 0.89) to C-12 (*δ*_C_ 82.4)/C-13 (*δ*_C_ 44.9)/C-14 (*δ*_C_ 44.4)/C-17 (*δ*_C_ 53.1), from CH_3_-19 (*δ*_H_ 0.91) to C-1 (*δ*_C_ 37.1)/C-5 (*δ*_C_ 141.1)/C-9 (*δ*_C_ 49.0)/C-10 (*δ*_C_ 36.9), from CH_3_-21 (*δ*_H_ 1.38)/H-20 (*δ*_H_ 2.00)/H-23a (*δ*_H_ 1.77)/H-26a (*δ*_H_ 3.53) to C-22 (*δ*_C_ 109.3) were observed. In addition, the ROESY correlations of H-12 (*δ*_H_ 3.88) with H-18 (*δ*_H_ 0.89) and H-20 (*δ*_H_ 2.00) indicated that the OH-12 was *α*-oriented (Fig. 3).

**Table 1:** ^1^H NMR spectroscopic data of compounds **1**–**5** (*δ* in ppm, *J* in Hz, C_5_D_5_N)

Position	**1**^a^	**2**^a^	**3**^a^	**4**^a^	**5**^b^
1a	1.73 (o)	1.75 (d, 3.6)	1.73 (o)	1.70 (o)	1.73 (m)
1b	1.07 (d, 3.8)	0.97 (s)	1.00 (s)	1.15 (o)	0.95 (m)
2a	1.99 (m)	2.07 (o)	2.21 (m)	2.18 (m)	2.06 (d)
2b	1.62 (m)	1.72 (d, 6.3)	1.86 (m)	1.89 (m)	1.86 (d)
3	3.58 (o)	3.69 (m)	3.95 (o)	4.03 (m)	3.87 (m)
4a	2.45 (m)	2.60 (o)	2.72 (o)	4.02 (m)	2.81 (o)
4b	2.29 (m)	2.45 (t, 12.3)	1.82 (o)	2.47 (m)	2.74 (o)
6	5.18 (o)	5.26 (o)	5.22 (d, 5.0)	10.22 (s)	5.35 (o)
7a	1.76 (o)	1.91 (o)	1.83 (m)		1.95 (o)
7b	1.73 (o)	1.60 (o)	1.47 (m)		1.58 (o)
8	1.44 (m)	1.51 (m)	1.49 (m)	2.67 (m)	1.61 (m)
9	1.87 (d, 3.8)	0.95 (d, 20.9)	0.91 (m)	1.03 (m)	0.98 (m)
11a	2.22 (m)	1.56 (m)	1.48 (m)	1.32 (o)	1.57 (2H, m)
11b	1.57 (m)	1.47 (m)		1.03 (o)	
12a	3.88 (br, s)	2.18 (m)	2.12 (m)	1.69 (m)	2.40 (m)
12b		1.91 (m)	1.83 (m)	1.03 (m)	1.60 (m)
14	1.56 (o)	2.04 (m)	2.03 (m)	1.36 (m)	1.50 (m)
15a	1.94 (o)	2.17 (m)	2.21 (m)	2.65 (m)	2.61 (m)
15b	1.44 (o)	1.51 (m)	1.56 (m)	2.01 (m)	2.41 (m)
16	4.42 (m)	4.43 (t, 6.9)	4.58 (t, 7.2)	4.59 (m)	
17	3.31 (dd, 8.6, 6.1)			1.80 (dd, 8.5, 6.1)	
18	0.89 (s)	0.93 (s)	1.14 (s)	0.88 (s)	0.94 (s)
19	0.91 (s)	0.97 (s)	1.00 (s)	0.83 (s)	1.10 (s)
20	2.00 (m)	2.20 (q)	3.39 (q, 7.2)	1.98 (m)	
21	1.38 (d, 7.0)	1.18 (d, 7.1)	1.31 (d, 7.2)	1.13 (d, 7.0)	2.34 (s)
23a	1.77 (o)	1.92 (m)	4.00 (m)	1.67 (m)	2.76 (m)
23b	1.38 (o)	1.52 (m)		1.58 (m)	2.72 (m)
24a	1.67 (m)	2.21 (m)	2.29 (o)	1.58 (m)	1.95 (m)
24b	1.28 (m)	1.86 (m)	2.21 (o)	1.24 (m)	1.51 (m)
25	1.55 (d, 6.2)	1.87 (m)	2.29 (m)	1.59 (o)	1.92 (m)
26a	3.53 (o)	4.12 (o)	3.99 (m)	4.90 (br s)	3.79 (m)
26b	3.46 (o)	3.92 (m)	3.90 (m)	3.54 (br s)	3.73 (m)
27a	0.67 (d, 5.4)	4.12 (o)	3.73 (m)	0.71 (d, 4.7z)	1.14 (d, 6.6)
27b		3.94 (m)	3.68 (m)		
16*'* 22*'*	3-Api	3-Api	3-Glc	3-Glc	7.12 (o) 7.07 (o) 3-Glc
1*'*	5.64 (d, 3.2)	5.72 (o)	4.92 (d, 7.1)	5.02 (d, 7.3)	4.96 (o)
2*'*	4.63 (o)	4.83 (m)	4.18 (o)	4.23 (m)	4.22 (m)
3*'*			4.18 (o)	4.20 (m)	4.22 (m)
4*'*a 4*'*b 5*'*a	4.51 (m) 4.25 (m) 4.62 (o)	4.48 (d, 9.3) 4.24 (d, 9.3) 4.13 (2H, o)	4.19 (o) 3.89 (o)	4.40 (m) 3.64 (m)	4.41 (m) 3.61 (m)
5*'*b	4.43 (o)				
6*'*a			4.74 (d, 5.5)	4.21 (o)	4.19 (o)
6*'*b			4.28 (d, 5.5)	4.05 (o)	4.05 (o)
	5*'*-Rha	2′-Rha	2′-Rha	2′-Rha	2′-Rha
1*''*	5.30 (br s)	5.85 (br s)	6.31 (br s)	6.44 (br s)	6.41 (br s)
2*''*	3.92 (m)	4.56 (o)	4.76 (o)	4.86 (m)	4.87 (m)
3*''*	4.08 (m)	4.70 (br, s)	4.58 (o)	4.62 (m)	4.67 (m)
4*''*	3.63 (m)	4.31 (o)	4.32 (o	4.36 (m)	4.38 (m)
5*''*	4.24 (m)	4.49 (m)	4.94 (m)	4.93 (m)	4.97 (m)
6*''*	1.55 (d, 6.2)	1.72 (d, 6.3)	1.75 (d, 6.1)	1.59 (d, 6.1)	1.60 (o)
	12-Glc		6*'*-Glc	4′-Rha	4′-Rha
1*'''*	4.86 (d, 7.8)		5.04 (d, 8.0)	5.82 (br s)	5.84 (br s)
2*'''*	4.06 (m)		4.01 (o)	4.54 (m)	4.52 (m)
3*'''*	4.23 (m)		4.18 (o)	4.54 (m)	4.54 (m)
4*'''*	4.25 (m)		4.12 (o)	4.44 (m)	4.45 (m)
5*'''*	3.97 (m)		4.18 (o)	4.92 (m)	4.94 (m)
6*'''*a 6*'''*b	4.53 (o) 4.39 (d, 5.0)		4.49 (d, 11.5) 4.33 (o)	1.59 (d, 6.1)	1.59 (o)
				4′′-Rha	4′′-Rha
1*''''*				6.28 (br s)	6.29 (br s)
2*''''*				4.90 (m)	4.90 (m)
3*''''*				4.54 (m)	4.52 (m)
4*''''*				4.33 (m)	4.31 (m)
5*''''*				4.94 (m)	4.37 (m)
6*''''*				1.73 (d, 6.2)	1.78 (d, 6.1)

*s* singlet, *d* doublet, *t* triplet, *q* quartet, *br* broad, *m* multiplet, *o* overlapped

^a^Measured at 500 MHz. ^b^Measured at 600 MHz

For the sugar part, the pentose was inferred as *β*-d-apiofuranoside by the ^13^C NMR signals at *δ*c 108.1 (d, C-1*'*), 78.4 (d, C-2*'*), 79.0 (s, C-3*'*), 74.8 (t, C-4*'*), and 72.9 (t, C-5*'*) with those of corresponding carbons of *α*- and *β*-d-apiofuranoside and *α*- and *β*-l-apiofuranoside [12, 13]. And the two hexose units were assigned to be a l-rhamnopyranosyl and a d-glucopyranosyl by their NMR data, the acid hydrolysis of **1**, and the HPLC analysis (retention time) of their L-cysteine methyl esters followed by conversion into *O*-tolyl isothiocyanate derivatives and the authentic samples’ derivatives. And the *β*-configuration of glucopyranosyl was revealed by the coupling constant (^3^*J*_1,2_ > 7.0 Hz) [14], while the anomeric configuration of rhamnopyranosyl was identified as *α*-orientated on the basis of the chemical shift values of C-3*''* (*δ*_C_ 72.9) and C-5*''* (*δ*_C_ 70.6) with those of corresponding carbons of methyl *α*- and *β*-rhamnopyranoside [15]. The sequence of the sugar chain at C-3 of the aglycone was established from the following HMBC corrletions: H-1*'* (*δ*_H_ 5.64) of Api with C-3 (*δ*_C_ 77.5) of the aglycone, H-1*''* (*δ*_H_ 5.30) of the Rha with C-5*'* (*δ*_C_ 72.9) of Api, and H-1*'''* (*δ*_H_ 4.86) of the Glc with C-12 (*δ*_C_ 82.4) of the aglycone (Fig. 2). Thus, the structure of **1** was elucidated as 12-*O-β*-d-glucopyranosy-(25*R*)-spirost-5-en-3*β*,12*β*-diol-3-*O*-*α*-l-rhamnopyranosyl-(1 → 5)-*β*-d-apiofuranoside, and named ypsilandroside U.

Compound **2** was isolated as an amorphous powder with a molecular formula of C_38_H_60_O_13_ determined by the positive-ion HRESI-MS at *m/z* 747.3921 [M + Na]^+^, (calcd. for C_38_H_60_O_13_Na, 747.3926) and ^13^C NMR data (Table 2). Its NMR spectra suggested that **2** is a spirostane saponin with a disaccharide chain. Comparison of the ^1^H and ^13^C NMR data of **2** (Tables 1 and 2) with those of ypsiparoside C obtained from the same genus [16] revealed that they shared the same aglycone. The two monosaccharides and their absolute configurations were determined as *β*-d-apiose and *α*-l-rhamnose by the same methods with compound **1**. The HMBC correlations from H-1*'* (*δ*_H_ 5.72) to C-3 (*δ*_C_ 77.7), and from H-1*''* (*δ*_H_ 5.85) of the rhamnopyransyl to C-2*'* (*δ*_C_ 82.4) established the sequence for 3-*O*-sugar chain as *O*-*α*-l-rhamnopyranosyl-(1 → 2)-*β*-d-apiofuranoside (Fig. 2). Therefore, the structure of **2** was determined as (25*R*)-spirost-5-en-3*β*,17*α*,27-triol-3-*O*-*α*-l-rhamnopyranosyl-(1 → 2)-*β*-d-apiofuranoside, and named ypsilandroside V.

**Table 2:** ^13^C NMR spectroscopic data of compounds **1**–**5** (*δ* in ppm, C_5_D_5_N)

Position	**1**^a^	**2**^a^	**3**^a^	**4**^a^	**5**^b^
1	37.1 (t)	37.6 (t)	37.6 (t)	36.3 (t)	37.2 (t)
2	30.2 (t)	30.3 (t)	30.3 (t)	29.9 (t)	30.0 (t)
3	77.5 (d)	77.7 (d)	76.8 (d)	77.7 (d)	77.8 (d)
4	39.3 (t)	39.3 (t)	39.1 (t)	30.7 (t)	38.9 (t)
5	141.1 (s)	140.8 (s)	140.9 (s)	169.5 (s)	140.9 (s)
6	121.6 (d)	121.9 (d)	121.7 (d)	189.3 (d)	121.7 (d)
7	32.0 (t)	32.4 (t)	32.4 (t)	139.6 (s)	31.9 (t)
8	31.8 (d)	32.3 (d)	32.3 (d)	45.8 (d)	30.8 (d)
9	49.0 (d)	50.2 (d)	50.1 (d)	60.4 (d)	50.4 (d)
10	36.9 (s)	37.1 (s)	37.1 (s)	46.5 (s)	37.0 (s)
11	27.6 (t)	20.9 (t)	20.9 (t)	20.8 (t)	21.2 (t)
12	82.4 (d)	32.1 (t)	32.4 (t)	40.1 (t)	36.8 (t)
13	44.9 (s)	45.1 (s)	45.8 (s)	43.3 (s)	47.1 (s)
14	44.4 (d)	53.0 (d)	53.1 (d)	54.3 (d)	57.6 (d)
15	32.1 (t)	31.8 (t)	31.9 (t)	35.3 (t)	32.3 (t)
16	81.0 (d)	90.2 (d)	90.8 (d)	81.3 (d)	140.6 (s)
17	53.1 (d)	90.1 (s)	90.1 (s)	62.4 (d)	151.8 (s)
18	17.0 (q)	17.2 (q)	17.4 (q)	16.8 (q)	16.4 (q)
19	19.3 (q)	19.5 (q)	19.4 (q)	15.5 (q)	19.2 (q)
20	42.2 (d)	45.3 (d)	38.8 (d)	41.9 (d)	131.1 (s)
21	15.3 (q)	9.6 (q)	9.4 (q)	15.1 (q)	14.6 (q)
22	109.3 (s)	110.5 (s)	112.7 (s)	109.2 (s)	139.9 (s)
23	32.0 (t)	27.5 (t)	68.1(d)	31.9 (t)	31.4 (t)
24	29.4 (t)	21.2 (t)	33.1 (t)	29.3 (t)	35.4 (t)
25	30.6 (d)	36.1 (d)	40.4 (d)	30.7 (d)	36.6 (d)
26	66.8 (t)	60.6 (t)	63.1 (t)	66.9 (t)	67.3 (t)
27 16*'* 22*'*	17.4 (q)	61.4 (t)	64.0 (t)	17.4 (q)	17.2 (q) 122.8 (d) 127.3 (d)
	3-Api	3-Api	3-Glc	3-Glc	3-Glc
1′	108.1 (d)	107.0 (d)	100.7 (d)	100.9 (d)	100.2 (d)
2′	78.4 (d)	82.4 (d)	77.5 (d)	77.9 (d)	77.9 (d)
3′	79.0 (s)	80.5 (s)	79.5 (d)	77.4 (d)	77.6 (d)
4′	74.8 (t)	74.9 (t)	71.6 (d)	77.7 (d)	77.6 (d)
5′	72.9 (t)	65.9 (t)	78.4 (d)	77.2 (d)	76.9 (d)
6′			69.9 (t)	61.3 (t)	61.1 (t)
	5*'*-Rha	2′-Rha	2′-Rha	2′-Rha	2′-Rha
1′′	102.8 (d)	102.0 (d)	102.0 (d)	101.9 (d)	102.1 (d)
2′′	72.4 (d)	72.7 (d)	72.6 (d)	72.5 (d)	72.6 (d)
3′′	72.9 (d)	72.0 (d)	72.8 (d)	72.9 (d)	72.8 (d)
4′′	74.3 (d)	74.0 (d)	74.2 (d)	74.2 (d)	74.0 (d)
5′′	70.6 (d)	70.3 (d)	69.5 (d)	69.5 (d)	69.5 (d)
6′′	18.6 (q)	18.7 (q)	18.7 (q)	18.5 (q)	18.3 (q)
	12-Glc		6*'*-Glc	4′-Rha	4′-Rha
1′′′	106.6 (d)		105.5 (d)	102.3 (d)	102.1 (d)
2′′′	75.6 (d)		75.2 (d)	72.9 (d)	72.8 (d)
3′′′	78.8 (d)		78.4 (d)	73.3 (d)	73.2 (d)
4′′′	71.9 (d)		71.6 (d)	80.4 (d)	80.3 (d)
5′′′	78.4 (d)		78.4 (d)	68.4 (d)	68.2 (d)
6′′′	63.1 (t)		62.7 (t)	18.6 (q)	18.8 (q)
				4′′-Rha	4′′-Rha
1′′′′				103.4 (d)	103.2 (d)
2′′′′				72.7 (d)	72.8 (d)
3′′′′				72.9 (d)	72.4 (d)
4′′′′				74.0 (d)	74.1 (d)
5′′′′				70.5 (d)	70.3 (d)
6′′′′				18.9 (q)	18.6 (q)

^a^Measured at 125 MHz. ^b^Measured at 150 MHz

Compound **3** was isolated as an amorphous powder and had a molecular formula of C_45_H_72_O_20_ as determined by the positive-ion HRESI-MS data (*m/z* 955.4505 [M + Na]^+^, calcd. for C_45_H_72_O_20_Na, 955.4509) and ^13^C NMR data (Table 2). Inspection of the NMR spectra (Tables 1 and 2) of **3** revealed that it possessed a spirotanol skeleton with a trisaccharide chain consisting of one rhamnopyranosyl and two glucopyranosyls. Comparing its ^1^H and ^13^C NMR data (Tables 1 and 2) with those of trillitschonide S6 [17] indicated that they shared the same aglycone. The *α*-orientations of OH-23 and CH_2_OH-25 were supported by the ROESY correlations between H-23 (*δ*_H_ 4.00) and H-20 (*δ*_H_ 3.39)/H-25 (*δ*_H_ 2.29) (Fig. 3). The absolute configurations and the anomeric configurations of monosaccharides were determined by the same methods with the above compounds. The sequence of the sugar chain at C-3 of the aglycone was established by the HMBC correlations from H-1*'* (*δ*_H_ 4.92) to C-3 (*δ*_C_ 76.8), from H-1*''* (*δ*_H_ 6.31) to C-2*'* (*δ*_C_ 77.5), and from H-1*'''* (*δ*_H_ 5.04) to C-6*'* (*δ*_C_ 69.9) (Fig. 2). Consequently, the structure of **3** was established as (23*S*,25*S*)-spirost-5-en-3*β*,17*α*,23,27-tetraol-3-*O*-*β*-d-glucopyranosyl-(1 → 6)-[*α*-l-rhamnopyranosyl-(1 → 2)]-*β*-d-glucopyranoside, and named ypsilandroside W.

Compound **4** possessed a molecular formula C_51_H_80_O_21_ determined by the HRESI-MS at *m/z* 1051.5077 [M + Na]^+^, (calcd. for C_51_H_80_O_21_Na, 1051.5084) and ^13^C NMR data (Table 2). The UV spectrum of **4** showed absorption maxima at 254.5 nm, suggesting the presence of a conjugated enal system. When comparing its ^1^H and ^13^C NMR data (Tables 1 and 2) with those of ypsilandroside H [10], it was suggested that they shared the same sugar sequence and the similar aglycone, except for the compound **4** has no hydroxyl substituent at the C-17. The above deduction could be verified by the HMBC correlations from H-21 (*δ*_H_ 1.13) and H-18 (*δ*_H_ 0.88) to C-17 (*δ*_C_ 62.4) and ^1^H‒^1^H COSY correlations between H-16 (*δ*_H_ 4.59) and H-17 (*δ*_H_ 1.80) (Fig. 2). The HMBC correlations from H-1*'* (*δ*_H_ 5.02) to C-3 (*δ*_C_ 77.7), from H-1*''* (*δ*_H_ 6.44) to C-2*'* (*δ*_C_ 77.9), from H-1*'''* (*δ*_H_ 5.82) to C-4*'* (*δ*_C_ 77.7), and from H-1*''''* (*δ*_H_ 6.28) to C-4*'''* (*δ*_C_ 80.4) confirmed that compound **3** had the same sequence of 3-*O*-sugar chain as that of ypsilandroside H (Fig. 2). Thus, the structure of **4** was elucidated as (25*R*)-B-nor(7)-6-carboxaldehyde-spirost-5(7)-en-3*β*-ol-3-*O*-*α*-l-rhamnopyranosyl-(1 → 4)-*α*-l-rhamnopyranosyl-(1 → 4)-[*α*-l-rhamnopyranosyl-(1 → 2)]-*β*-d-glucopyranoside, and named ypsilandroside X.

The molecular formula of compound **5** was determined as C_53_H_82_O_19_ by the HRESI-MS at *m/z* 1045.5352 [M + Na]^+^ (calcd. for C_53_H_82_O_19_Na, 1045.5343) and ^13^C NMR data (Table 2). Its NMR spectra indicated that compound **5** was a cholestane tetraglycosides containing an aromatic ring. Analysis of the ^1^H and ^13^C NMR data (Tables 1 and 2) of **5** suggested that it was similar to that of parispseudoside A [18], and the major difference was the absence of a glucopyranosyl group at OH-26 site. With the assistance of HSQC experiment, ^1^H and ^13^C NMR data (Tables 1 and 2) showed four anomeric protons at *δ*_H_ 4.96 (o, H-1*'*), 6.41 (br s, H-1*''*), 5.84 (br s, H-1*'''*), and 6.29 (s, H-1*''''*) and their corresponding anomeric carbons at *δ*_C_ 100.2 (C-1*'*), 102.1 (C-1*''*), 102.1 (C-1*'''*), and 103.2 (C-1*''''*). The sequence of sugar units was consistent with that of compound **4** by HMBC experiment (Fig. 2). As a result, the structure of **5** was assigned as homo-aro-cholest-5-en-3*β*,26-diol-3-*O*-*α*-l-rhamnopyranosyl-(1 → 4)-*α*-l-rhamnopyranosyl-(1 → 4)-[*α*-l-rhamnopyranosyl-(1 → 2)]-*β*-d-glucopyranoside, and named ypsilandroside Y.

Because the whole plants of *Y.* *thibetica* has been used in folk medicine for treatment of uterine bleeding and traumatic hemorrhage in China, the isolated compounds (**1**–**5**) were evaluated for their induced platelet aggregation activity and ADP (adenosine diphosphate) was used as a positive control. Unfortunately, the results showed all isolated saponins did not exhibit the inducing platelet aggregation activity at the tested concentration of 100 μM.

## Experimental section

### General experimental procedures

Optical rotations were measured by a JASCO P-1020 polarimeter (Jasco Corp., Japan). UV spectra were recorded on a Shimadzu UV2401 PC spectrophotometer (Shimadzu Corp., Japan). HRESI-MS was recorded on an Agilent 1290 UPLC/6540 Q-TOF mass spectrometer (Agilent Corp., USA). The NMR experiments were performed on Bruker AVANCE III 500, Avance III-600, and AV 800 spectrometers (Bruker Corp., Switzerland). Silica gel (200–300 mesh, Qingdao Marine Chemical Co., Ltd., People’s Republic of China), RP-18 (50 μm, Merck, Germany), and Sephadex LH-20 (Pharmacia, Stockholm, Sweden) were used for column chromatography (CC). An Agilent 1260 system (Agilent Corp., America) with a Zorbax SB-C18 column (5 μm, 9.4 × 250 mm) was used for HPLC separation. TLC was carried out on silica gel HSGF_254_ plates (Qingdao Marine Chemical Co., China) or RP-18 F_254_ (Merck, Darmstadt, Germany).

### Plant material

The whole plant materials of *Y.* *thibetica* were collected in August 2010 from Zhaotong City, Yunnan Provence, China, and identified by Prof. Xin-Qi Chen, Institute of Botany, Chinese Academy of Sciences, Beijing. A voucher specimen was deposited at the State Key Laboratory of Phytochemistry and Plant Resources in West China, Kunming Institute of Botany, Chinese Academy of Sciences.

### Extraction and isolation

The dried whole plants of *Y.* *thibetica* (110 kg) were crushed and extracted three times with 70% EtOH under reflux for a 3 h, 2 h and 2 h. Then, the combined extract was concentrated under reduced pressure. The crude extract (30 kg) was passed through YWD-3F macroporous resin and eluted successively with H_2_O, 40% EtOH, 75% EtOH, and 95% EtOH, respectively. Evaporated 75% EtOH fraction (crude saponin-rich mixture, 10 kg) was subjected to a silica gel column chromatography (CHCl_3_–MeOH, 20:1 → 8:2, v/v) to give eleven fractions (Fr. A–Fr. K). Fr. C (560 g) was subjected to a silica gel column chromatography (CHCl_3_–MeOH, 20:1 → 1:1, v/v) to give 14 fractions (Fr. C-1–Fr. C-14). Fr. C-11 (80 mg) was submitted to Sephadex LH-20 (MeOH) and chromatographically separated on an RP-18 column eluted with MeOH–H_2_O (40:60 → 70:30, v/v) and purified by preparative HPLC (MeCN–H_2_O, 40:60 → 50:50, v/v) to afford saponin **2** (*t*_R_ = 12.8 min, 10 mg). Fr. C-13 (45 g) was submitted to Sephadex LH-20 (MeOH) to give three subfractions (C-13–1–C-13–3). Subsequently, Fr. C-13–1 (150 mg) was further purified by preparative HPLC (MeCN–H_2_O, 25:75 → 35:65, v/v) to afford saponins **5** (*t*_R_ = 10.8 min, 7 mg) and **4** (*t*_R_ = 11.9 min, 12 mg), whereas saponins **3** (*t*_R_ = 11.1 min, 10 mg) and **1** (*t*_R_ = 14.8 min, 9 mg) were obtained from Fr. C-13–3 (208 mg) by preparative HPLC (MeCN–H_2_O, 30:70 → 45:55, v/v).

### Physical and spectroscopic data of new glycosides

#### Ypsilandroside U (*1*)

Amorphous solid; $[\alpha]_{D}^{18.6}$‒55.80 (*c* 0.20, MeOH); ^1^H (500 MHz, pyridine *d*_*5*_) and ^13^C (125 MHz, pyridine *d*_*5*_) NMR data, see Tables 1 and 2; HRESIMS *m/z* 893.4500 [M + Na]^+^ (calcd. for C_44_H_70_O_17_Na, 893.4505) (Additional file 1).

#### Ypsilandroside V (*2*)

Amorphous solid; $[\alpha]_{D}^{18.6}$‒190.00 (*c* 0.12, MeOH); ^1^H (500 MHz, pyridine-*d*_*5*_) and ^13^C (125 MHz, pyridine-*d*_*5*_) NMR data, see Tables 1 and 2; HRESIMS *m/z* 747.3921 ([M + Na]^+^, calcd. for C_38_H_60_O_13_Na, 747.3926) (Additional file 1).

#### Ypsilandroside W (*3*)

Amorphous solid; $[\alpha]_{D}^{20.5}$‒125.67 (*c* 0.12, MeOH); ^1^H (500 MHz, pyridine-*d*_*5*_) and ^13^C (125 MHz, pyridine-*d*_*5*_) NMR data, see Tables 1 and 2; HRESIMS *m/z* 955.4505 [M + Na]^+^ (calcd. for C_45_H_72_O_20_Na, 955.4509) (Additional file 1).

#### Ypsilandroside X (*4*)

Amorphous solid; $[\alpha]_{D}^{18.6}$‒106.40 (*c* 0.15, MeOH); UV (MeOH) λ_max_ (log *ε*) 202.5 (3.9), 254.5 (3.9) nm; ^1^H (500 MHz, pyridine-*d*_*5*_) and ^13^C (125 MHz, pyridine-*d*_*5*_) NMR data, see Tables 1 and 2; HRESIMS *m/z* 1051.5077 [M + Na]^+^ (calcd. for C_51_H_80_O_21_Na, 1051.5084) (Additional file 1).

#### Ypsilandroside Y (*5*)

Amorphous solid; $[\alpha]_{D}^{18.6}$‒48.18 (*c* 0.11, MeOH); UV (MeOH) λ_max_ (log *ε*) 203 (4.5) nm; ^1^H (600 MHz, pyridine-*d*_*5*_) and ^13^C (150 MHz, pyridine-*d*_*5*_) NMR data, see Tables 1 and 2; HRESIMS *m/z* 1045.5352 [M + Na]^+^ (calcd. for C_53_H_82_O_19_Na, 1045.5343) (Additional file 1).

### Acid hydrolysis of compounds 1–5 and determination of the absolute configuration of the sugars by HPLC

Compounds **1**‒**5** (1.0 mg each) in 6 M CF_3_COOH (1,4-dioxane-H_2_O 1:1, 1.0 mL) were heated at 99 ℃ for 2 h, respectively. The reaction mixture was diluted with H_2_O (1.0 mL) and then extracted with EtOAc (3 × 2.0 mL). Next, each aqueous layer was evaporated to dryness using rotary evaporation. Each dried residue was dissolved in pyridine (1.0 mL) mixed with l-cysteine methyl ester hydrochloride (1.0 mg) (Aldrich, Japan) and heated at 60 °C for 1 h. Then, *O*-tolyl isothiocyanate (5.0 μL) (Tokyo Chemical Industry Co., Ltd., Japan) was added to the mixture, this being heated at 60 °C for 1 h. Each reaction mixture was directly analyzed by reversed phase HPLC following the above procedure. Each reaction mixture was directly analyzed by analytical HPLC on a Poroshell 120 SB-C18 column (100 × 4.6 mm, 2.7 μm, Agilent) using an elution of CH_3_CN‒H_2_O (20:75 → 40:60, v/v) at a flow rate of 0.6 mL/min. As a result, the sugars in the test compounds were identified as d-glucose and l-rhamnose, respectively, by comparing their molecular weight and retention time with the standards (*t*_R_ 13.90 min for d-glucose; *t*_R_ 17.72 min for l-rhamnose).

### Platelet aggregation assays

Turbidometric measurements of platelet aggregation of the samples were performed in a Chronolog Model 700 Aggregometer (Chronolog Corporation, Havertown, PA, USA) according to Born’s method [19, 20]. Rabbit platelet aggregation study was completed within 3.0 h of preparation of platelet-rich plasma (PRP). Immediately after preparation of PRP, 250 μL was incubated in each test tube at 37 °C for 5.0 min and then 2.5 μL of compounds (100 μM) were individually added. The changes in absorbance as a result of platelet aggregation were recorded. The extent of aggregation was estimated by the percentage of maximum increase in light transmittance, with the buffer representing 100% transmittance. ADP (adenosine diphosphate) was used as a positive control with a 59.5 ± 6.1% maximal platelet aggregation rate at a concentration of 10 μM. 1% DMSO was used as a blank control with a 2.7 ± 0.6% maximal platelet aggregation. Data counting and analysis was done on SPSS 16.0, with experimental results expressed as mean ± standard error.

## Conclusion

Phytochemical reinvestigation on the whole plants of *Y.* *thibetica* obtained four new spirostanol glycosides, named ypsilandrosides U-X (**1**–**4**), and one new cholestanol glycoside, named ypsilandroside Y (**5**). Their structures have been illustrated by extensive spectroscopic data and chemical methods. Among them, compound **4** is a rare spirostanol glycoside which possesses a novel 5(6 → 7) abeo-steroidal aglycone, while compound **1** is a first spirostanol bisdesmoside attached to C-3 and C-12, respectively, obtained from the *Ypsilandra* species. This investigation enriched the cognition of the chemical constituents in *Y.* *thibetica.* Unfortunately, the bioassay results showed the five new saponins have no the activity of inducing platelet aggregation.

## Supplementary Information

**Additional file 1: Fig. S1.** ^1^H NMR spectrum (500 MHz) of compound **1** in pyridine-*d*_5_. **Fig. S2.** ^13^C NMR spectrum (125 MHz) of compound **1** in pyridine-*d*_5_. **Fig. S3.** ^1^H–^1^H COSY spectrum of compound **1** in pyridine-*d*_5_. **Fig. S4.** HSQC spectrum of compound **1** in pyridine-*d*_5_. **Fig. S5.** HMBC spectrum of compound **1** in pyridine-*d*_5_. **Fig. S6.** ROESY spectrum of compound **1** in pyridine-*d*_5_. **Fig. S7.** HRESI (+) MS spectrum of compound **1**. **Fig. S8.** ^1^H NMR spectrum (500 MHz) of compound **2** in pyridine-*d*_5_. **Fig. S9.** ^13^C NMR spectrum (125 MHz) of compound **2** in pyridine-*d*_5_. **Fig. S10.** ^1^H–^1^H COSY spectrum of compound **2** in pyridine-*d*_5_. **Fig. S11.** HSQC spectrum of compound **2** in pyridine-*d*_5_. **Fig. S12.** HMBC spectrum of compound **2** in pyridine-*d*_5_. **Fig. S13.** ROESY spectrum of compound **2** in pyridine-d5. **Fig. S14.** HRESI (+) MS spectrum of compound **2**. **Fig. S15.** ^1^H NMR spectrum (500 MHz) of compound **3** in pyridine-*d*_5_. **Fig. S16.** ^13^C NMR spectrum (125 MHz) of compound **3** in pyridine-*d*_5_. **Fig. S17.** ^1^H–^1^H COSY spectrum of compound **3** in pyridine-*d*_5_. **Fig. S18.** HSQC spectrum of compound **3** in pyridine-*d*_5_. **Fig. S19.** HMBC spectrum of compound **3** in pyridine-*d*_5_. **Fig. S20.** ROESY spectrum of compound **3** in pyridine-*d*_5_. **Fig. S21.** HRESI (+) MS spectrum of compound **3**. **Fig. S22** ^1^H NMR spectrum (500 MHz) of compound **4** in pyridine-*d*_5_.**Fig. S23.** ^13^C NMR spectrum (125 MHz) of compound **4** in pyridine-*d*_5_. **Fig. S24.** ^1^H–^1^H COSY spectrum of compound **4** in pyridine-*d*_5_. **Fig. S25.** HSQC spectrum of compound **4** in pyridine-*d*_5_. **Fig. S26.** HMBC spectrum of compound **4** in pyridine-*d*_5_. **Fig. S27.** ROESY spectrum of compound **4** in pyridine-*d*_5_. **Fig. S28.** HRESI (+) MS spectrum of compound **4**. **Fig. S29.** UV spectrum of compound **4**. **Fig. S30.** ^1^H NMR spectrum (600 MHz) of compound **5** in pyridine-*d*_5_. **Fig. S31.** ^13^C NMR spectrum (150 MHz) of compound **5** in pyridine-*d*_5_. **Fig. S32.** ^1^H–^1^H COSY spectrum of compound **5** in pyridine-*d*_5_. **Fig. S33.** HSQC spectrum of compound **5** in pyridine-*d*_5_. **Fig. S34.** HMBC spectrum of compound **5** in pyridine-*d*_5_. **Fig. S35.** ROESY spectrum of compound **5** in pyridine-*d*_5_. **Fig. S36**. HRESI (+) MS spectrum of compound **5**. **Fig. S37**. UV spectrum of compound **5**.
