# Supplementary material for: Ypsilandrosides U-Y, five new steroidal saponins from Ypsilandrathibetica
Source: Nat Prod Bioprospect. 2022 May 5;12(1):17. doi: 10.1007/s13659-022-00337-0 (PMC9068850; doi:10.1007/s13659-022-00337-0)
Supplement: Supplementary file 1 — Additional file 1: Fig. S1. 1H NMR spectrum (500 MHz) of compound 1 in pyridine-d5. Fig. S2. 13C NMR spectrum (125 MHz) of compound 1 in pyridine-d5. Fig. S3. 1H–1H COSY spectrum of compound 1 in pyridine-d5. Fig. S4. HSQC spectrum of compound 1 in pyridine-d5. Fig. S5. HMBC spectrum of compound 1 in pyridine-d5. Fig. S6. ROESY spectrum of compound 1 in pyridine-d5. Fig. S7. HRESI (+) MS spectrum of compound 1. Fig. S8. 1H NMR spectrum (500 MHz) of compound 2 in pyridine-d5. Fig. S9. 13C NMR spectrum (125 MHz) of compound 2 in pyridine-d5. Fig. S10. 1H–1H COSY spectrum of compound 2 in pyridine-d5. Fig. S11. HSQC spectrum of compound 2 in pyridine-d5. Fig. S12. HMBC spectrum of compound 2 in pyridine-d5. Fig. S13. ROESY spectrum of compound 2 in pyridine-d5. Fig. S14. HRESI (+) MS spectrum of compound 2. Fig. S15. 1H NMR spectrum (500 MHz) of compound 3 in pyridine-d5. Fig. S16. 13C NMR spectrum (125 MHz) of compound 3 in pyridine-d5. Fig. S17. 1H–1H COSY spectrum of compound 3 in pyridine-d5. Fig. S18. HSQC spectrum of compound 3 in pyridine-d5. Fig. S19. HMBC spectrum of compound 3 in pyridine-d5. Fig. S20. ROESY spectrum of compound 3 in pyridine-d5. Fig. S21. HRESI (+) MS spectrum of compound 3. Fig. S22 1H NMR spectrum (500 MHz) of compound 4 in pyridine-d5.Fig. S23. 13C NMR spectrum (125 MHz) of compound 4 in pyridine-d5. Fig. S24. 1H–1H COSY spectrum of compound 4 in pyridine-d5. Fig. S25. HSQC spectrum of compound 4 in pyridine-d5. Fig. S26. HMBC spectrum of compound 4 in pyridine-d5. Fig. S27. ROESY spectrum of compound 4 in pyridine-d5. Fig. S28. HRESI (+) MS spectrum of compound 4. Fig. S29. UV spectrum of compound 4. Fig. S30. 1H NMR spectrum (600 MHz) of compound 5 in pyridine-d5. Fig. S31. 13C NMR spectrum (150 MHz) of compound 5 in pyridine-d5. Fig. S32. 1H–1H COSY spectrum of compound 5 in pyridine-d5. Fig. S33. HSQC spectrum of compound 5 in pyridine-d5. Fig. S34. HMBC spectrum of compound 5 in pyridine-d5. Fig. S35. ROESY spectrum of compound 5 in [file 13659_2022_337_MOESM1_ESM.docx]

**Supplementary Material for**

**Ypsilandrosides U-Y, five new steroidal saponins from** ***Ypsilandra thibetica***

Wen-Tao Gao ^1,2,†^, Ling-Ling Yu ^2,3, †^, Jing Xie ^2,3^, Long-Gao Xiao ^2,3^, Shi-Juan Zhang ^2,3^, Wen-Yi Ma ^2,3^, Huan Yan ^2^, Hai-Yang Liu ^2,^*

^1^ *College of Traditional Chinese Medicine, Yunnan University of Chinese Medicine, Kunming 650500, China*

^2^ *State Key Laboratory of Phytochemistry and Plant Resources in West China, and Yunnan Key Laboratory of Natural Medicinal Chemistry, Kunming Institute of Botany, Chinese Academy of Sciences, Kunming 650201, China*

^3^ *University of Chinese Academy of Sciences, Beijing, 100049, China*

*Corresponding author.

E-mail addresses: haiyangliu@mail.kib.ac.cn (H.-Y. Liu)

^†^ These authors contributed equally to this work.

**Table of Contents**

**[Fig. S1](#_Toc88685194)**^[1](#_Toc88685194)^[H NMR spectrum (500 MHz) of compound](#_Toc88685194) **[1](#_Toc88685194)** [in pyridine-](#_Toc88685194)*[d](#_Toc88685194)*_[5](#_Toc88685194)_[. 3](#_Toc88685194)

[**Fig. S2** ^13^C NMR spectrum (125 MHz) of compound **1** in pyridine-*d*_5_. 11](#_Toc88685195)

[**Fig. S3** ^1^H–^1^H COSY spectrum of compound **1** in pyridine-*d*_5_. 12](#_Toc88685196)

[**Fig. S4** HSQC spectrum of compound **1** in pyridine-*d*_5_. 13](#_Toc88685197)

[**Fig. S5** HMBC spectrum of compound **1** in pyridine-*d*_5_. 14](#_Toc88685198)

[**Fig. S6** ROESY spectrum of compound **1** in pyridine-*d*_5_. 15](#_Toc88685199)

[**Fig. S7** HRESI (+) MS spectrum of compound **1**. 16](#_Toc88685200)

**[Fig. S8](#_Toc88685194)**^[1](#_Toc88685194)^[H NMR spectrum (500 MHz) of compound](#_Toc88685194) **[2](#_Toc88685194)** [in pyridine-](#_Toc88685194)*[d](#_Toc88685194)*_[5](#_Toc88685194)_[. 10](#_Toc88685194)

[**Fig. S9** ^13^C NMR spectrum (125 MHz) of compound **2** in pyridine-*d*_5_. 11](#_Toc88685195)

[**Fig. S10** ^1^H–^1^H COSY spectrum of compound **2** in pyridine-*d*_5_. 12](#_Toc88685196)

[**Fig. S11** HSQC spectrum of compound **2** in pyridine-*d*_5_. 13](#_Toc88685197)

[**Fig. S12** HMBC spectrum of compound **2** in pyridine-*d*_5_. 14](#_Toc88685198)

[**Fig. S13** ROESY spectrum of compound **2** in pyridine-*d*_5_. 15](#_Toc88685199)

[**Fig. S14** HRESI (+) MS spectrum of compound **2**. 16](#_Toc88685200)

[**Fig. S15** ^1^H NMR spectrum (500 MHz) of compound **3** in pyridine-*d*_5_. 17](#_Toc88685194)

[**Fig. S16** ^13^C NMR spectrum (125 MHz) of compound **3** in pyridine-*d*_5_. 18](#_Toc88685195)

[**Fig. S17** ^1^H–^1^H COSY spectrum of compound **3** in pyridine-*d*_5_. 19](#_Toc88685196)

[**Fig. S18** HSQC spectrum of compound **3** in pyridine-*d*_5_. 20](#_Toc88685197)

[**Fig. S19** HMBC spectrum of compound **3** in pyridine-*d*_5_. 21](#_Toc88685198)

[**Fig. S20** ROESY spectrum of compound **3** in pyridine-*d*_5_. 22](#_Toc88685199)

[**Fig. S21** HRESI (+) MS spectrum of compound **3**. 23](#_Toc88685200)

**[Fig. S22](#_Toc88685212)**^[1](#_Toc88685212)^[H NMR spectrum (500 MHz) of compound](#_Toc88685212) **[4](#_Toc88685212)** [in pyridine-](#_Toc88685212)*[d](#_Toc88685212)*_[5](#_Toc88685212)_[. 24](#_Toc88685212)

[**Fig. S23** ^13^C NMR spectrum (125 MHz) of compound **4** in pyridine-*d*_5_. 25](#_Toc88685213)

[**Fig. S24** ^1^H–^1^H COSY spectrum of compound **4** in pyridine-*d*_5_. 26](#_Toc88685214)

[**Fig. S25** HSQC spectrum of compound **4** in pyridine-*d*_5_. 27](#_Toc88685215)

[**Fig. S26** HMBC spectrum of compound **4** in pyridine-*d*_5_. 28](#_Toc88685216)

[**Fig. S27** ROESY spectrum of compound **4** in pyridine-*d*_5_. 29](#_Toc88685217)

[**Fig. S28** HRESI (+) MS spectrum of compound **4**. 30](#_Toc88685218)

[**Fig. S29** UV spectrum of compound **4**. 31](#_Toc88685219)

[**Fig. S30** ^1^H NMR spectrum (600 MHz) of compound **5** in pyridine-*d*_5_. 32](#_Toc88685203)

[**Fig. S31** ^13^C NMR spectrum (150 MHz) of compound **5** in pyridine-*d*_5_. 33](#_Toc88685204)

[**Fig. S32** ^1^H–^1^H COSY spectrum of compound **5** in pyridine-*d*_5_. 34](#_Toc88685205)

[**Fig. S33** HSQC spectrum of compound **5** in pyridine-*d*_5_. 35](#_Toc88685206)

[**Fig. S34** HMBC spectrum of compound **5** in pyridine-*d*_5_. 36](#_Toc88685207)

[**Fig. S35** ROESY spectrum of compound **5** in pyridine-*d*_5_. 37](#_Toc88685208)

[**Fig. S36** HRESI (+) MS spectrum of compound **5**. 38](#_Toc88685209)

[**Fig. S37** UV spectrum of compound **5**. 39](#_Toc88685210)


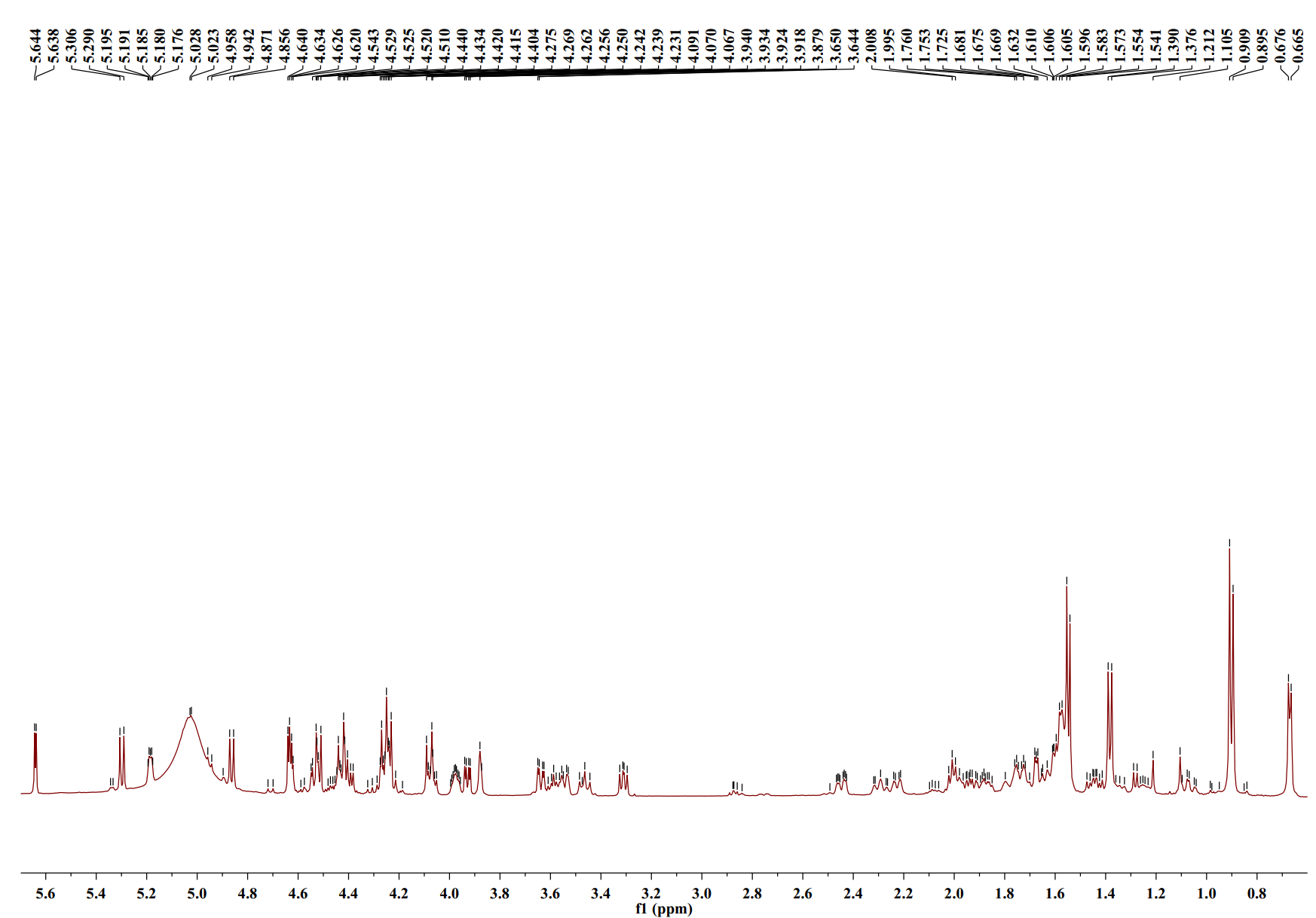


**Fig. S1** ^1^H NMR spectrum (500 MHz) of compound **1** in pyridine-*d*_5_.


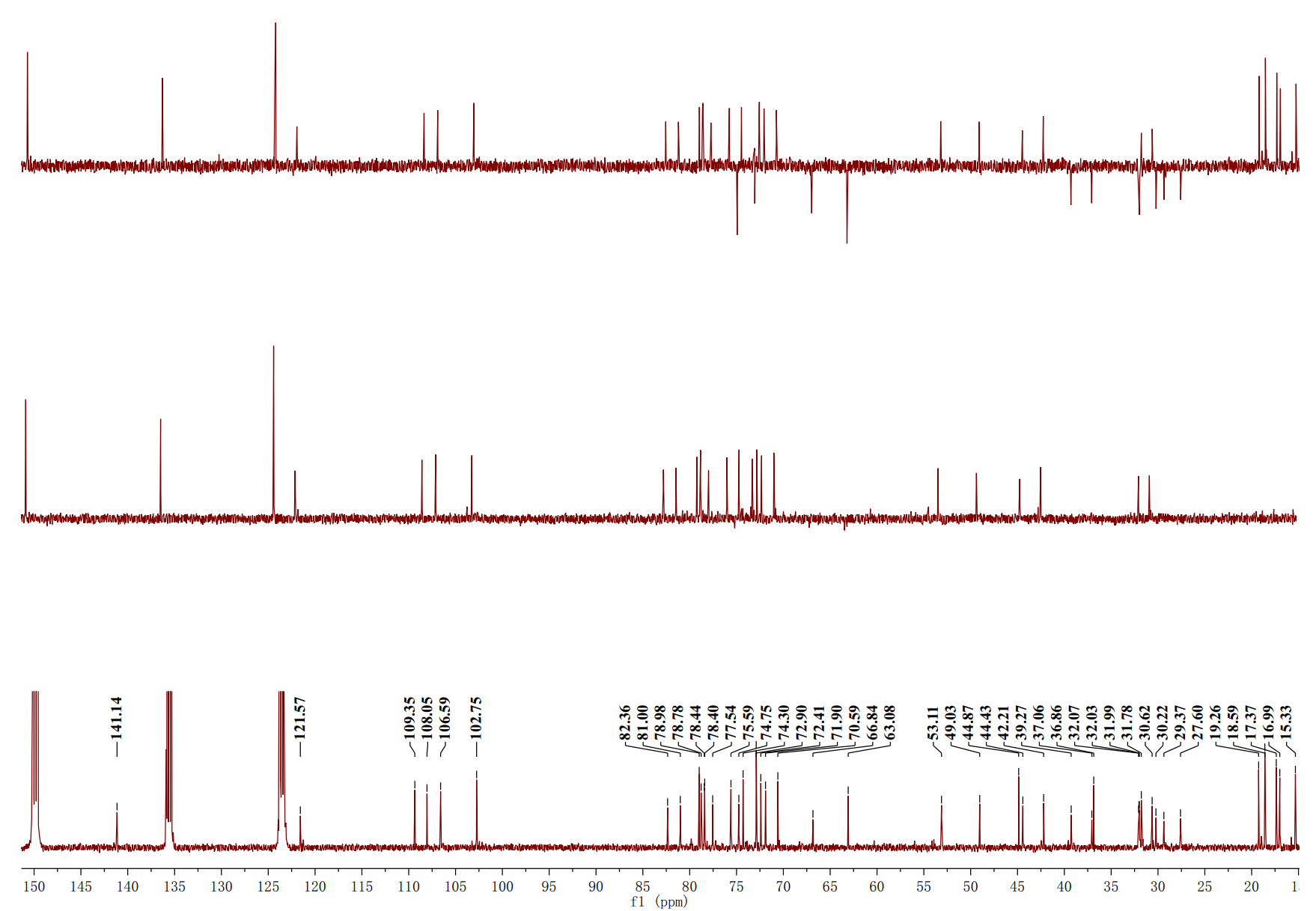


**Fig. S2** ^13^C NMR spectrum (125 MHz) of compound **1** in pyridine-*d*_5_.


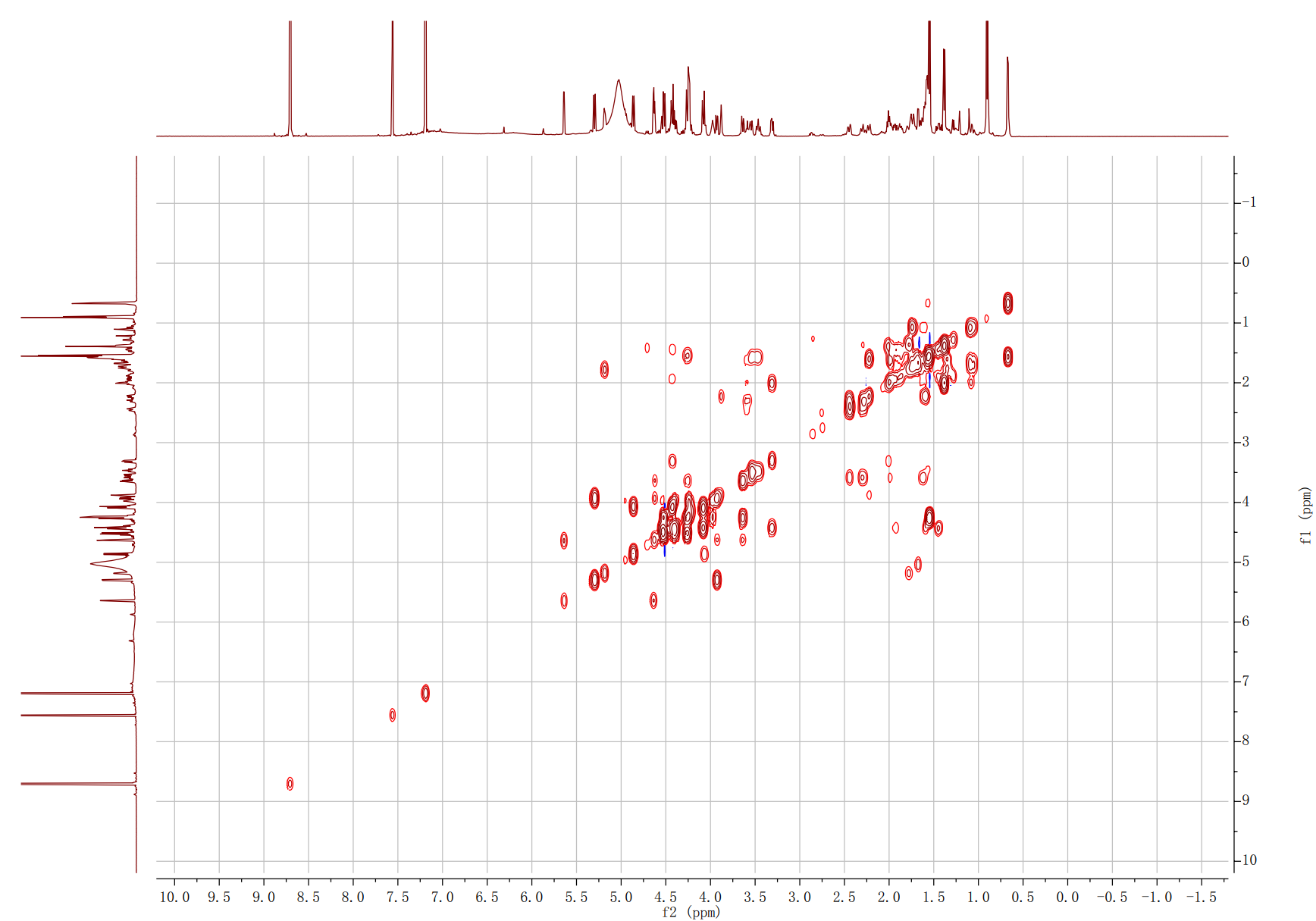


**Fig. S3** ^1^H–^1^H COSY spectrum of compound **1** in pyridine-*d*_5_.


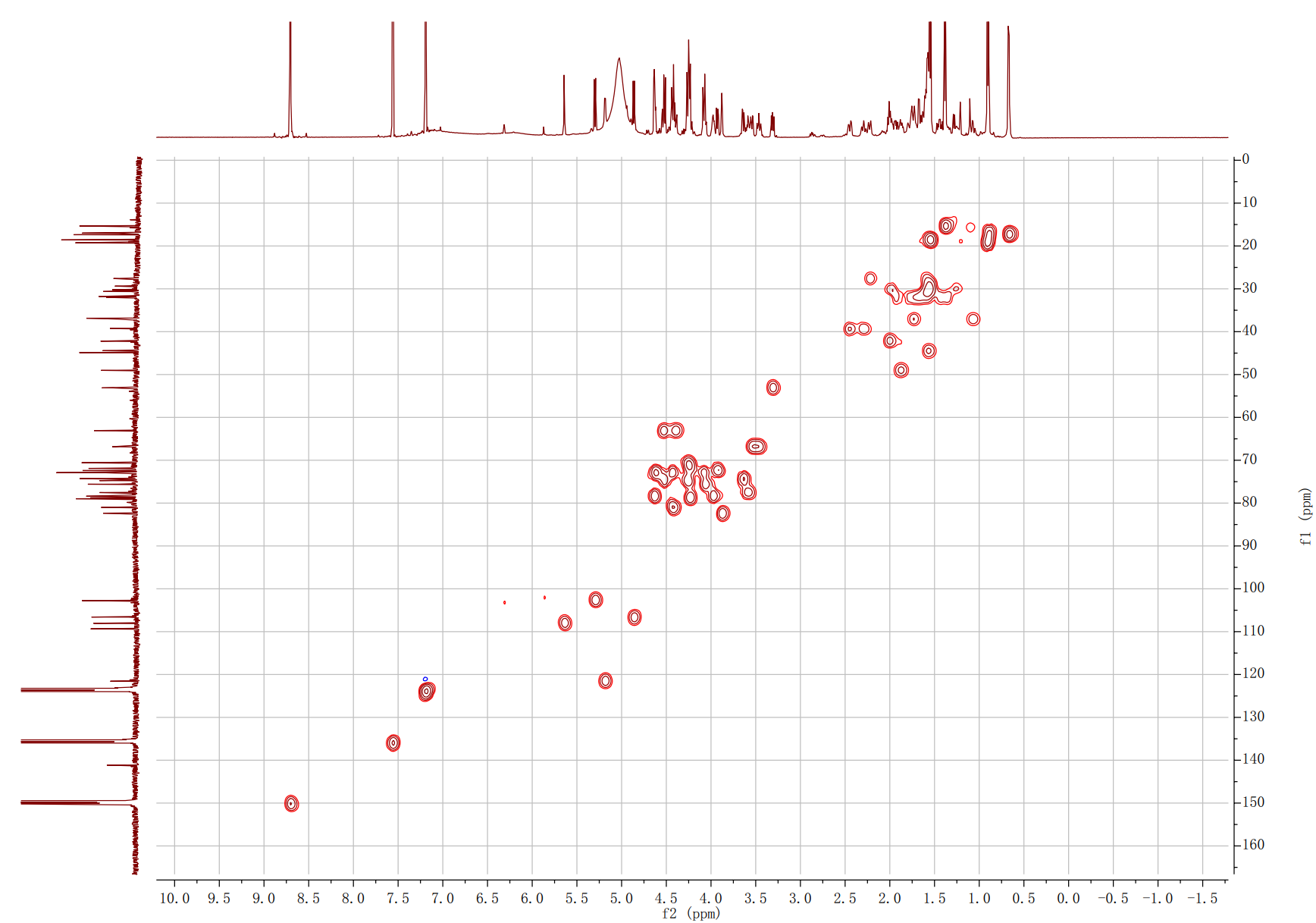


**Fig. S4** HSQC spectrum of compound **1** in pyridine-*d*_5_.


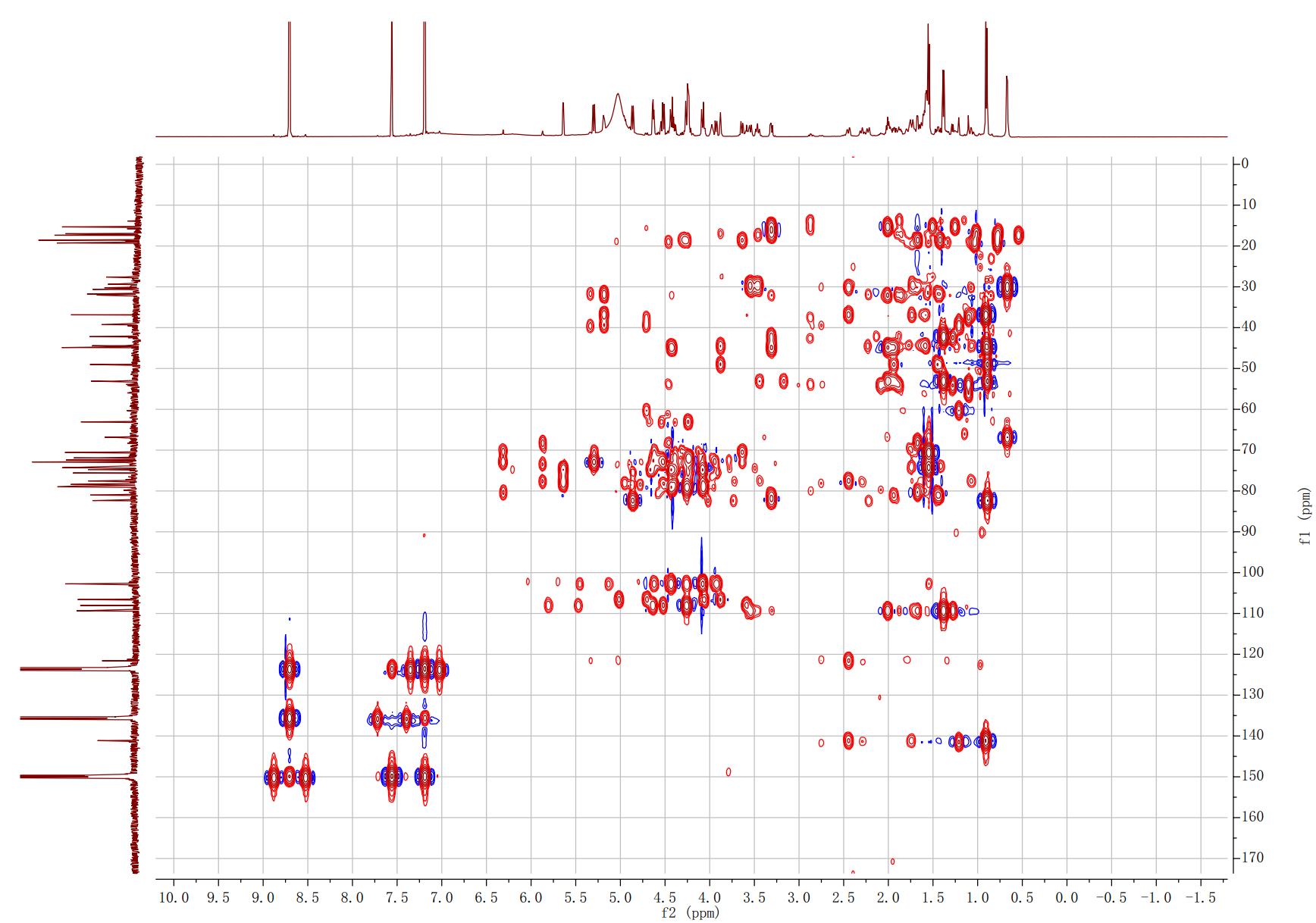


**Fig. S5** HMBC spectrum of compound **1** in pyridine-*d*_5_.


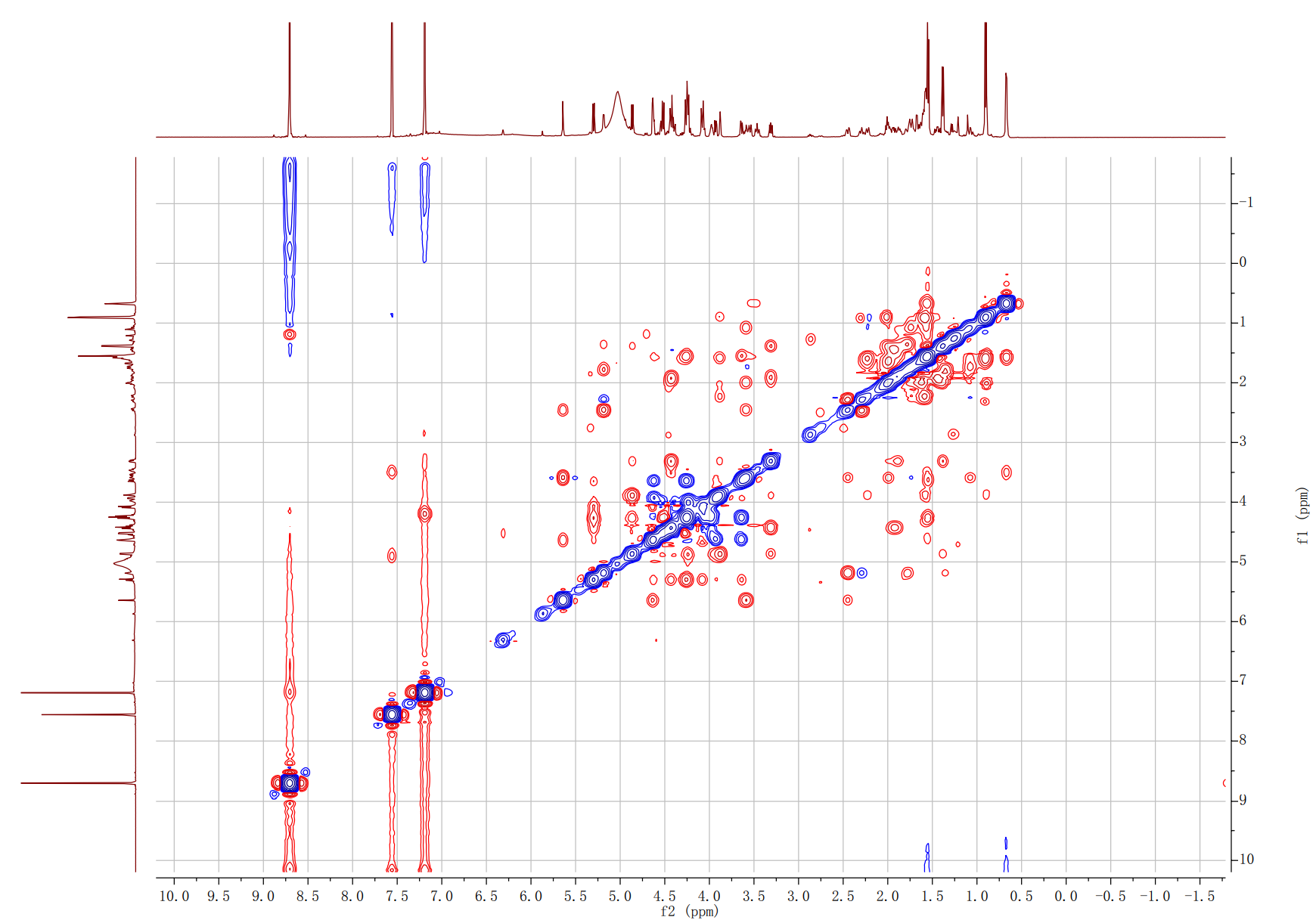


**Fig. S6** ROESY spectrum of compound **1** in pyridine-*d*_5_.

_
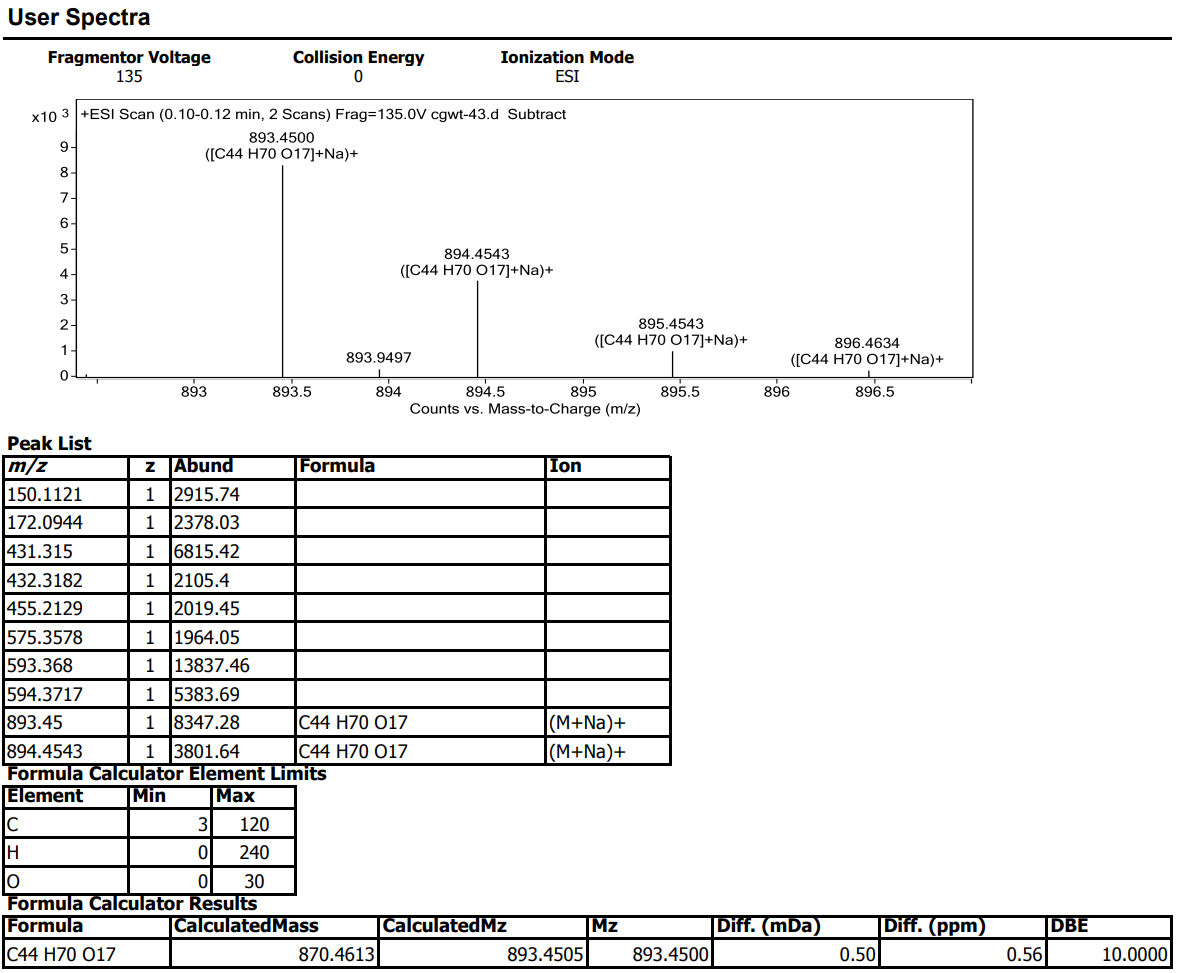
_

**Fig. S7** HRESI (+) MS spectrum of compound **1**.
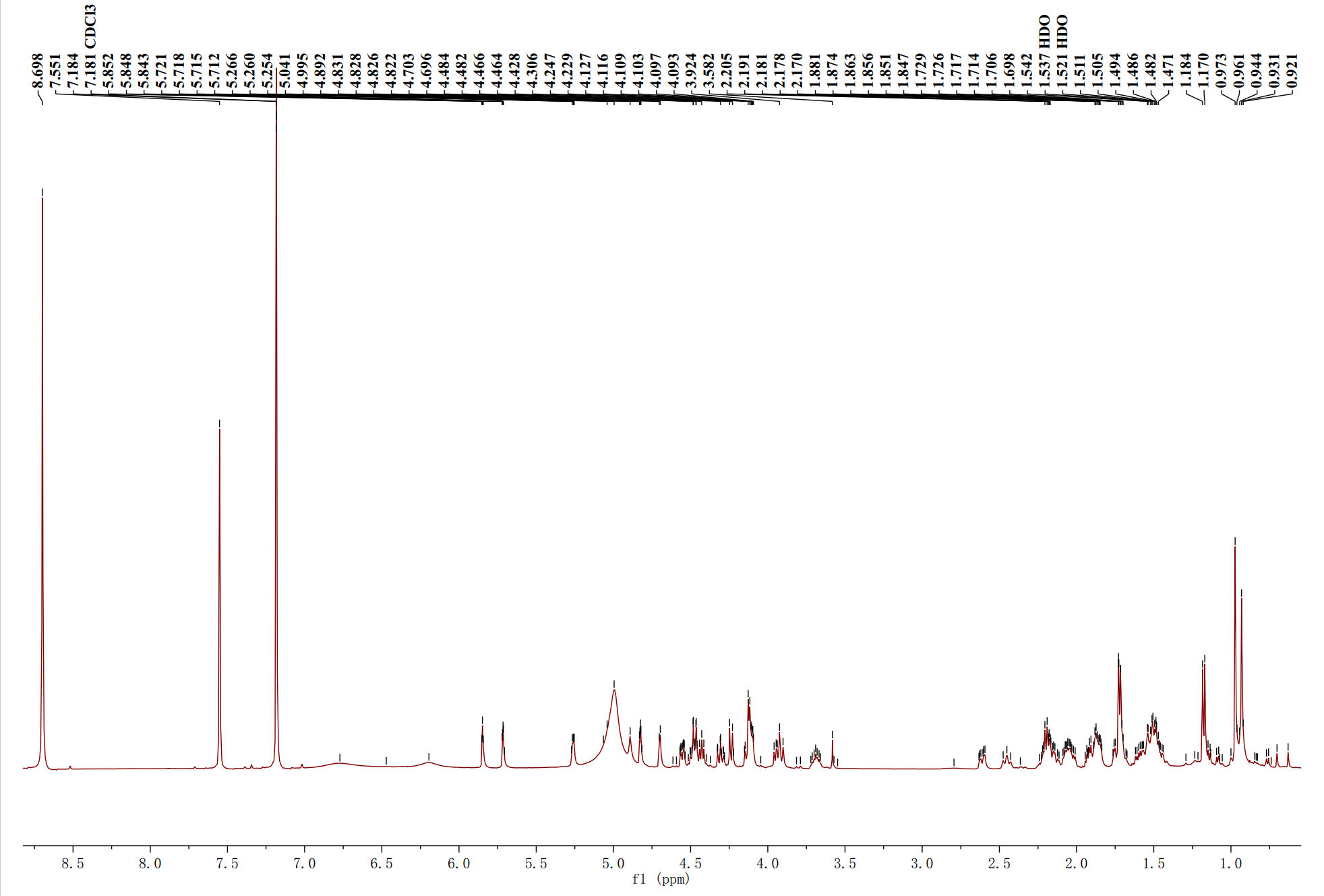


# **Fig. S8** ^1^H NMR spectrum (500 MHz) of compound **2** in pyridine-*d*_5_.


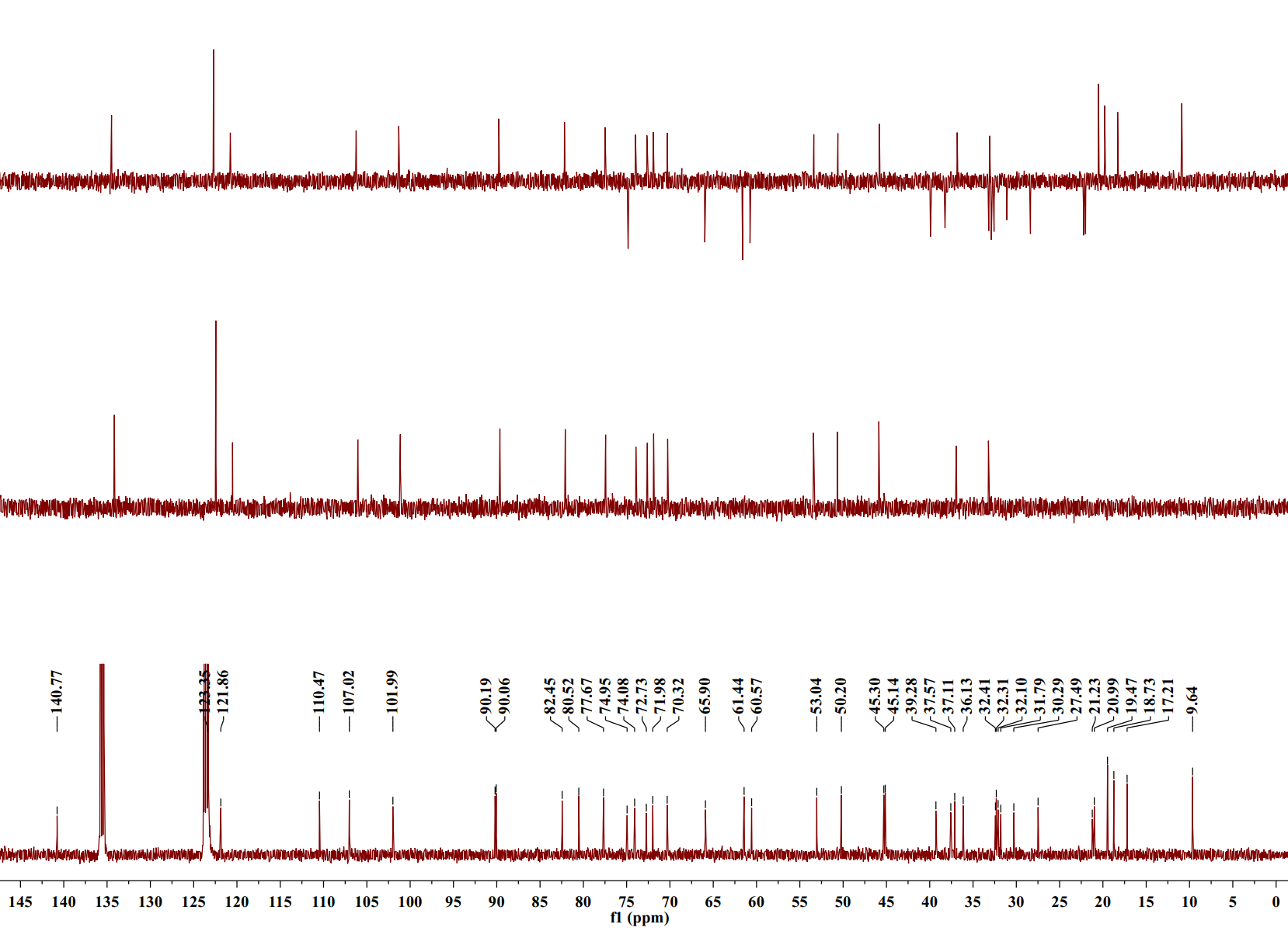


# **Fig. S9** ^13^C NMR spectrum (125 MHz) of compound **2** in pyridine-*d*_5_.


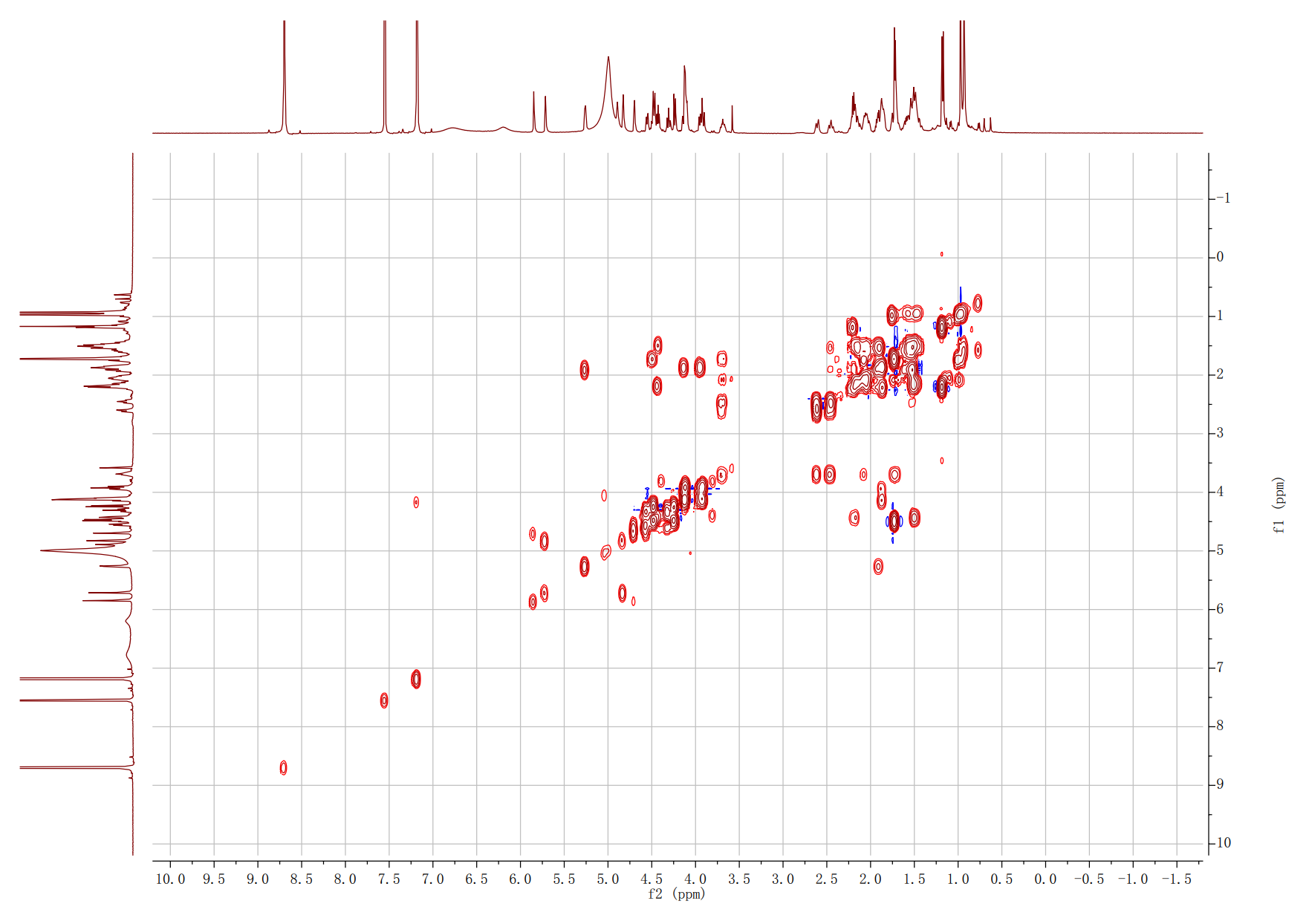


# **Fig. S10** ^1^H–^1^H COSY spectrum of compound **2** in pyridine-*d*_5_.


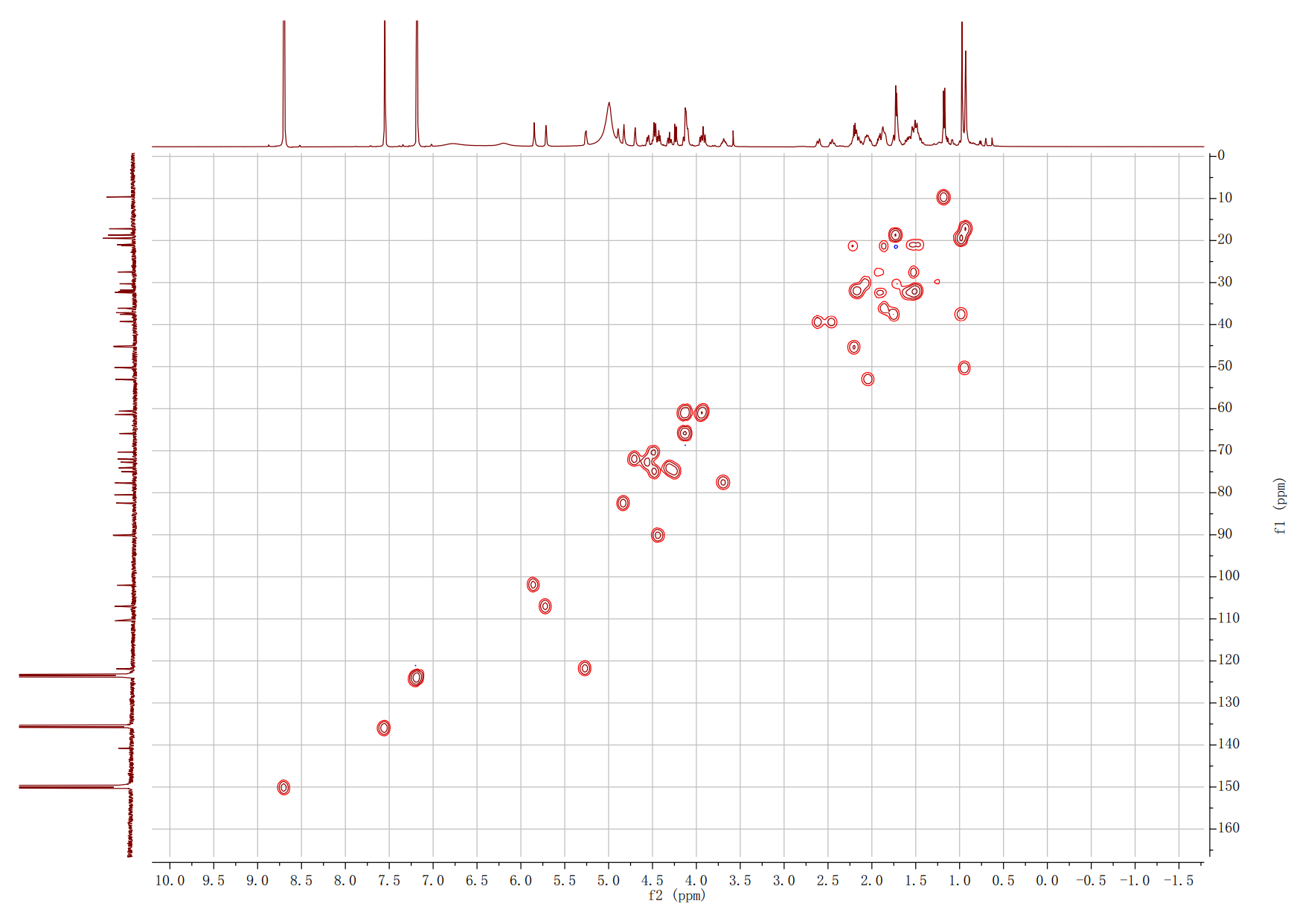


# **Fig. S11** HSQC spectrum of compound **2** in pyridine-*d*_5_.


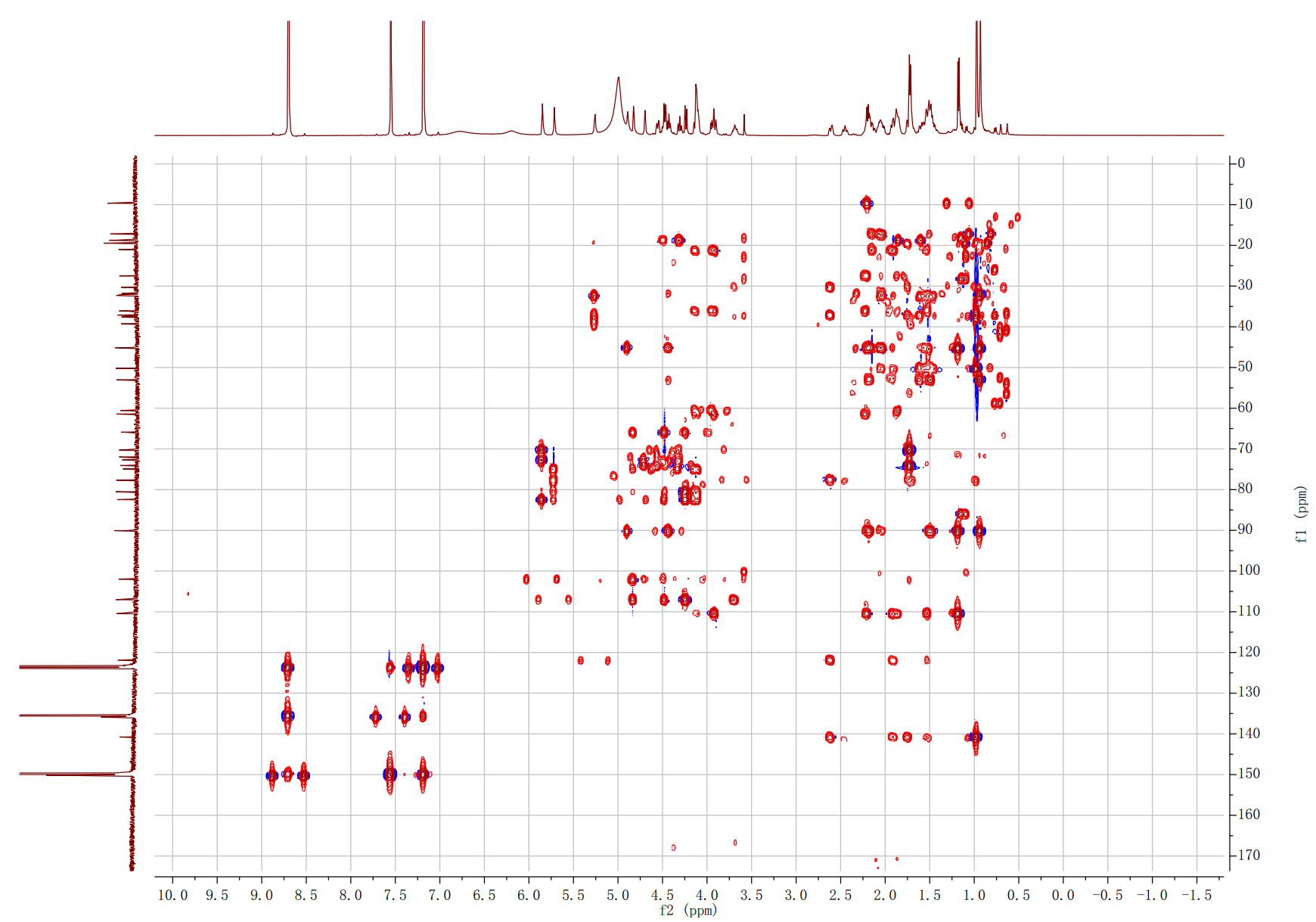


# **Fig. S12** HMBC spectrum of compound **2** in pyridine-*d*_5_.


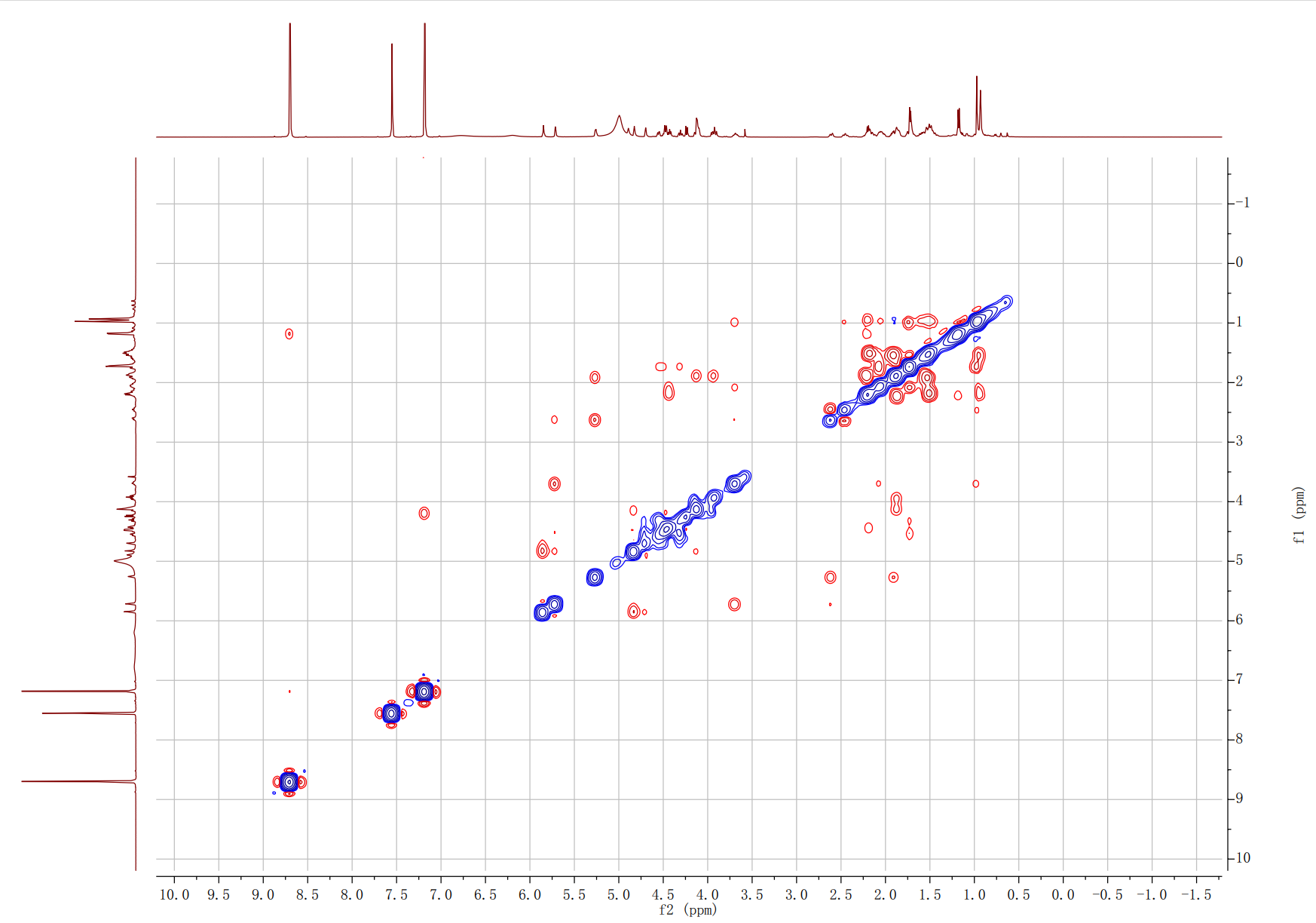


# **Fig. S13** ROESY spectrum of compound **2** in pyridine-*d*_5_.


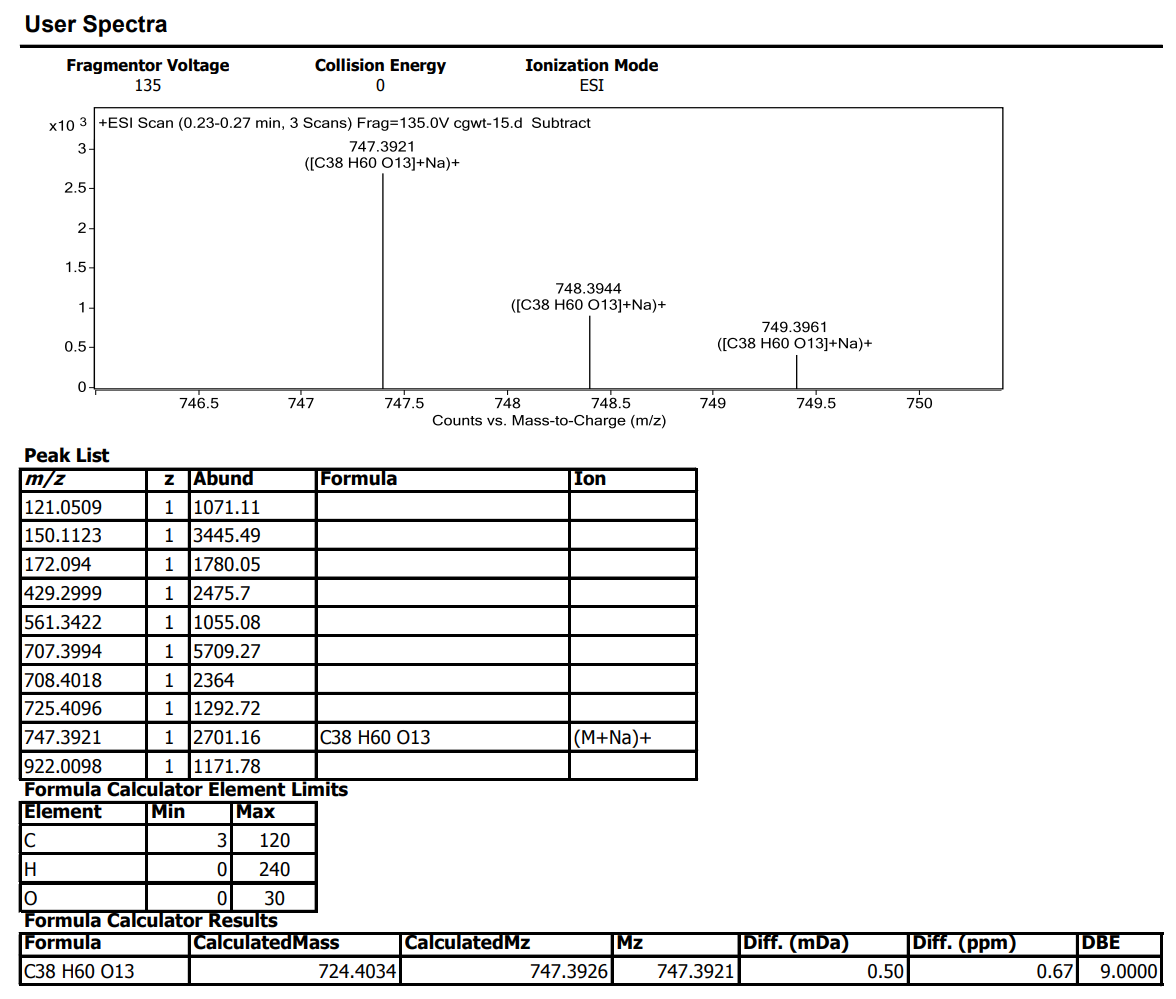


# **Fig. S14** HRESI (+) MS spectrum of compound **2**.

_
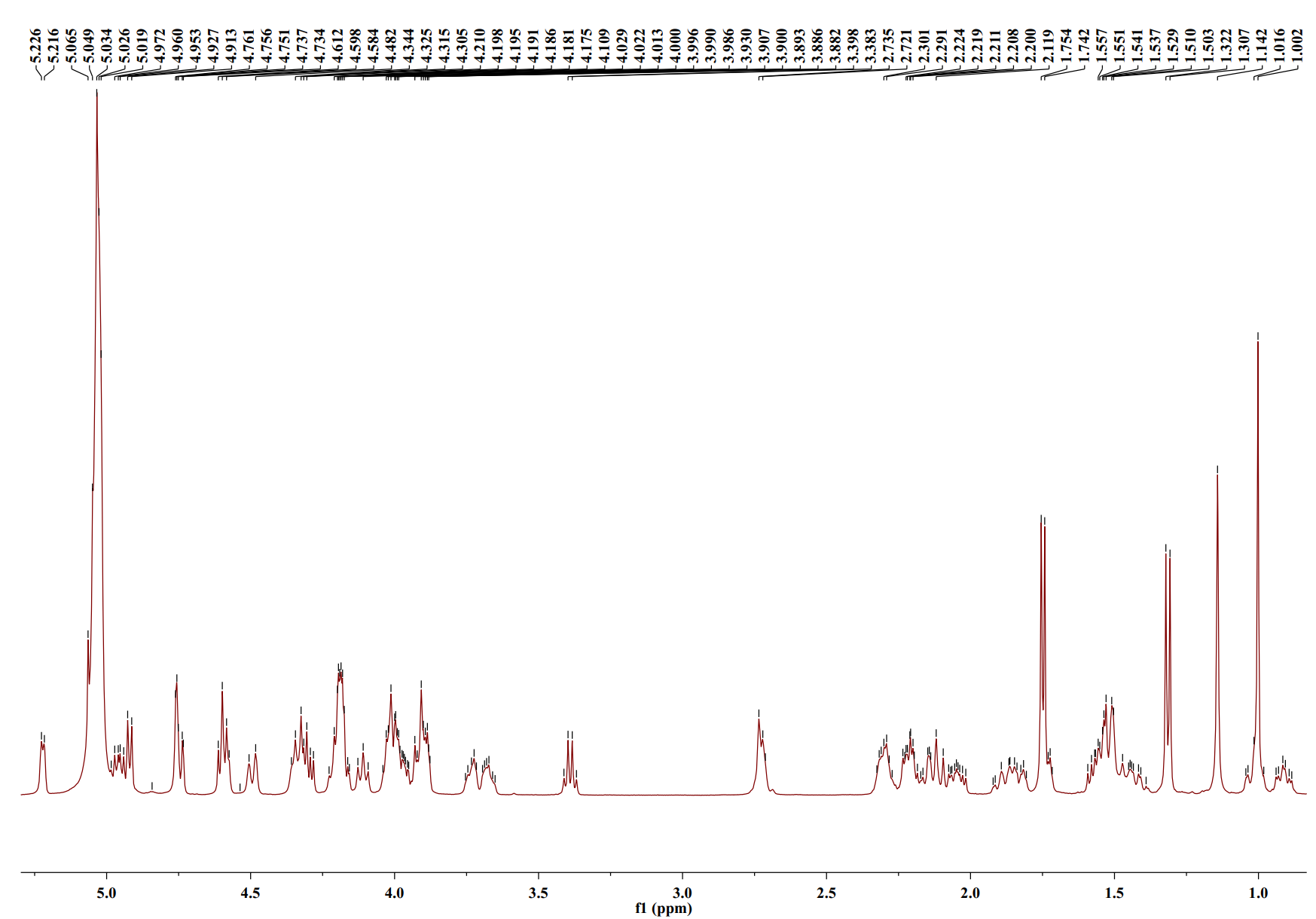
_

# **Fig. S15** ^1^H NMR spectrum (500 MHz) of compound **3** in pyridine-*d*_5_.


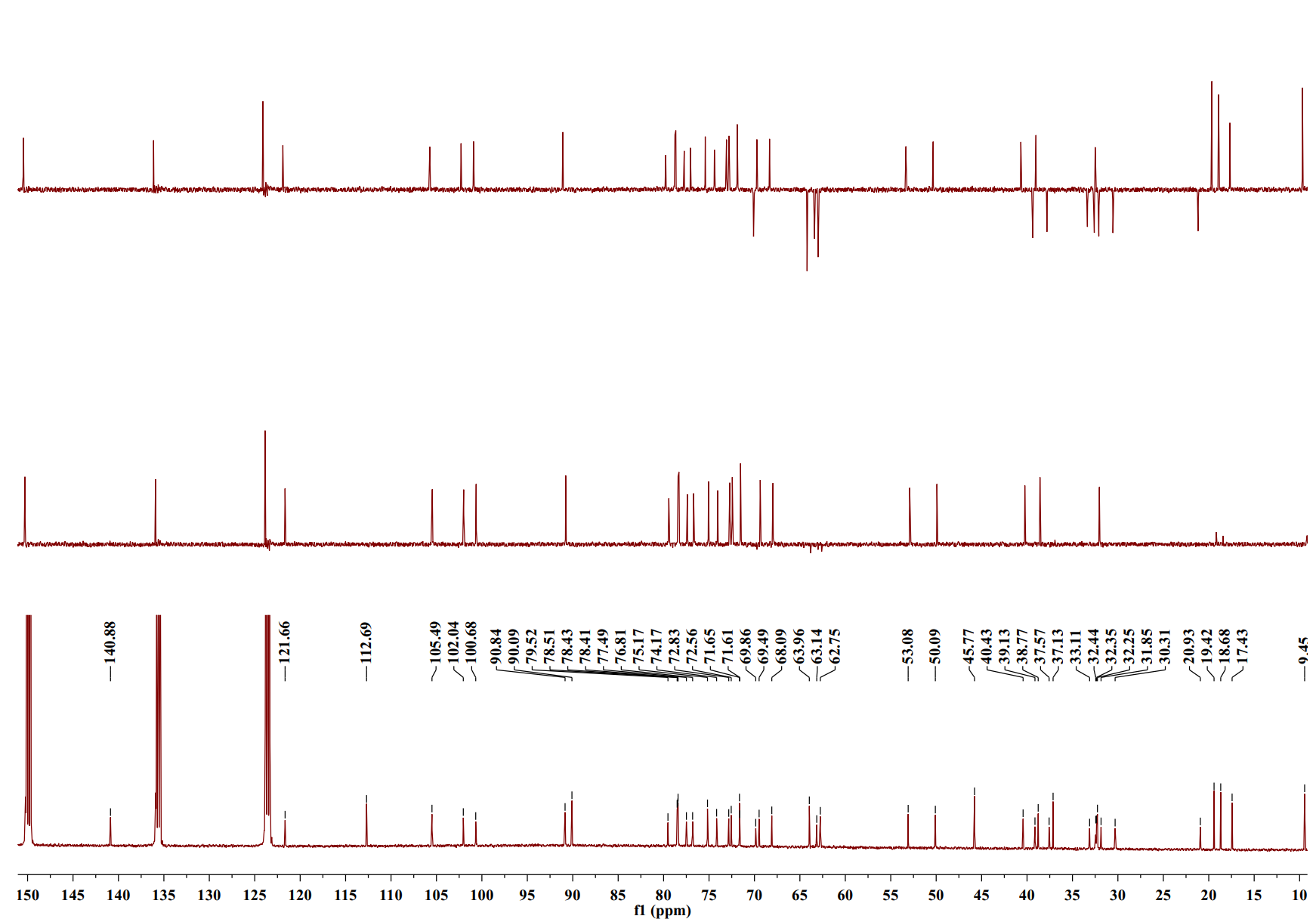


# **Fig. S16** ^13^C NMR spectrum (125 MHz) of compound **3** in pyridine-*d*_5_.


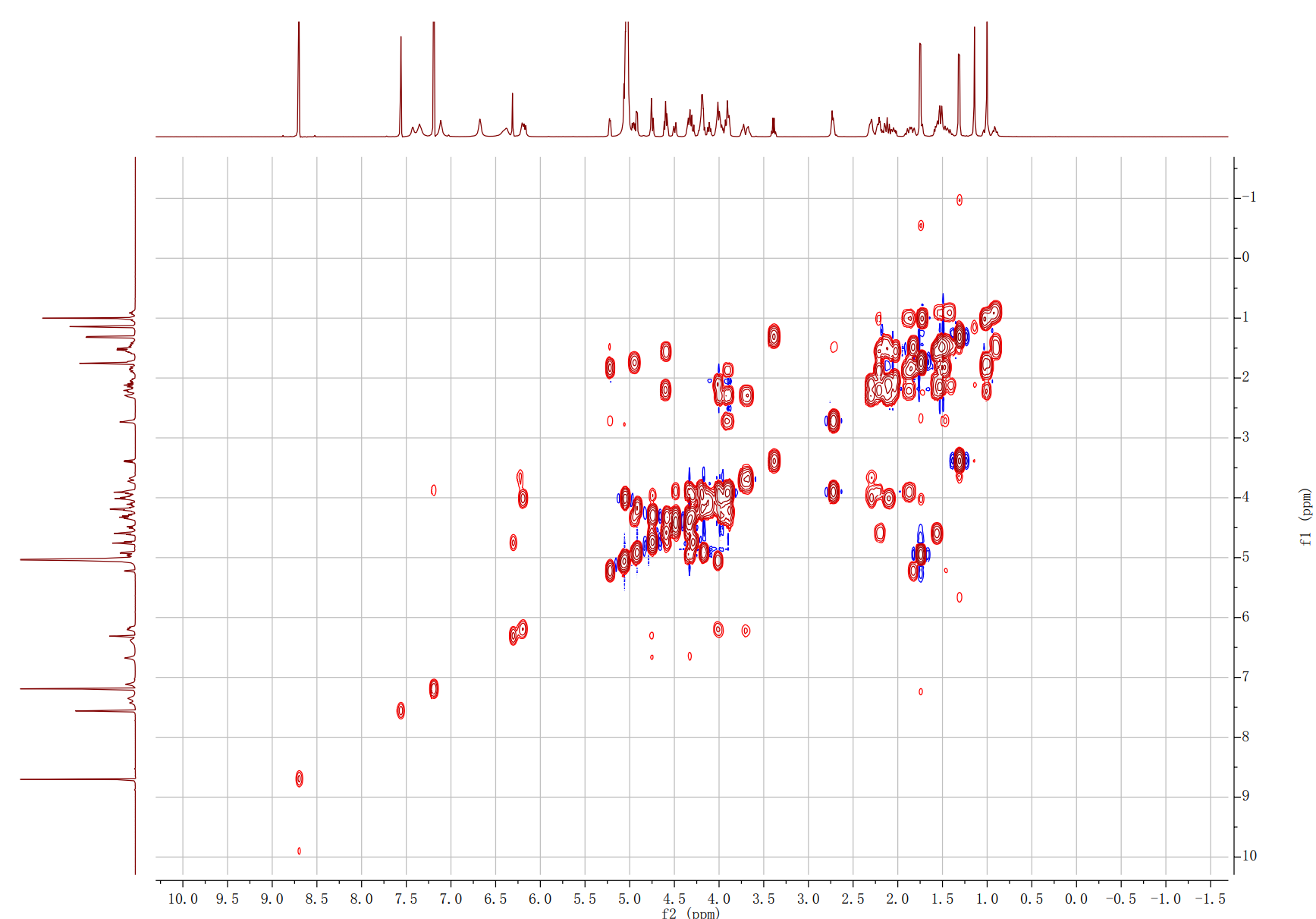


# **Fig. S17** ^1^H–^1^H COSY spectrum of compound **3** in pyridine-*d*_5_.


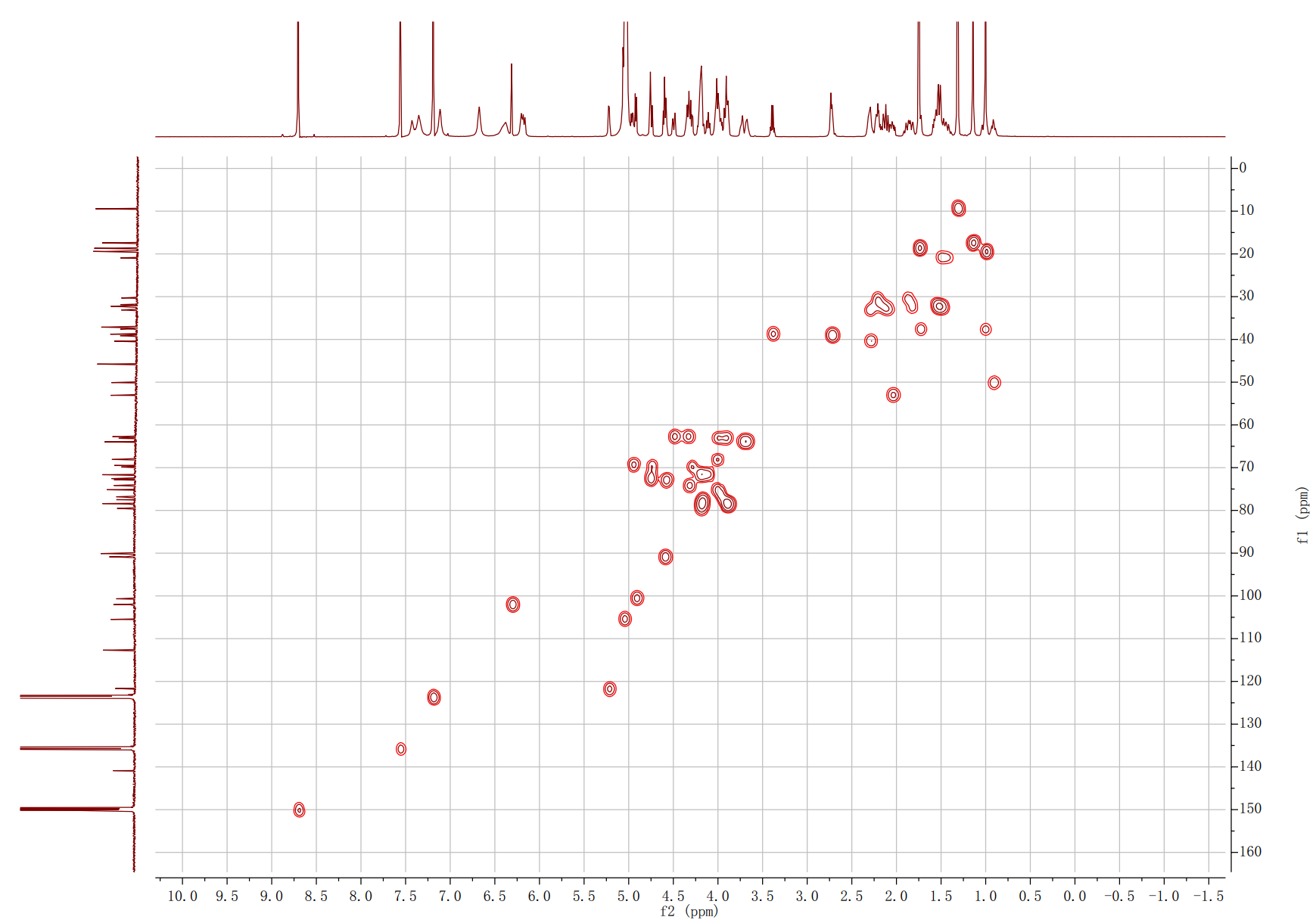


# **Fig. S18** HSQC spectrum of compound **3** in pyridine-*d*_5_.


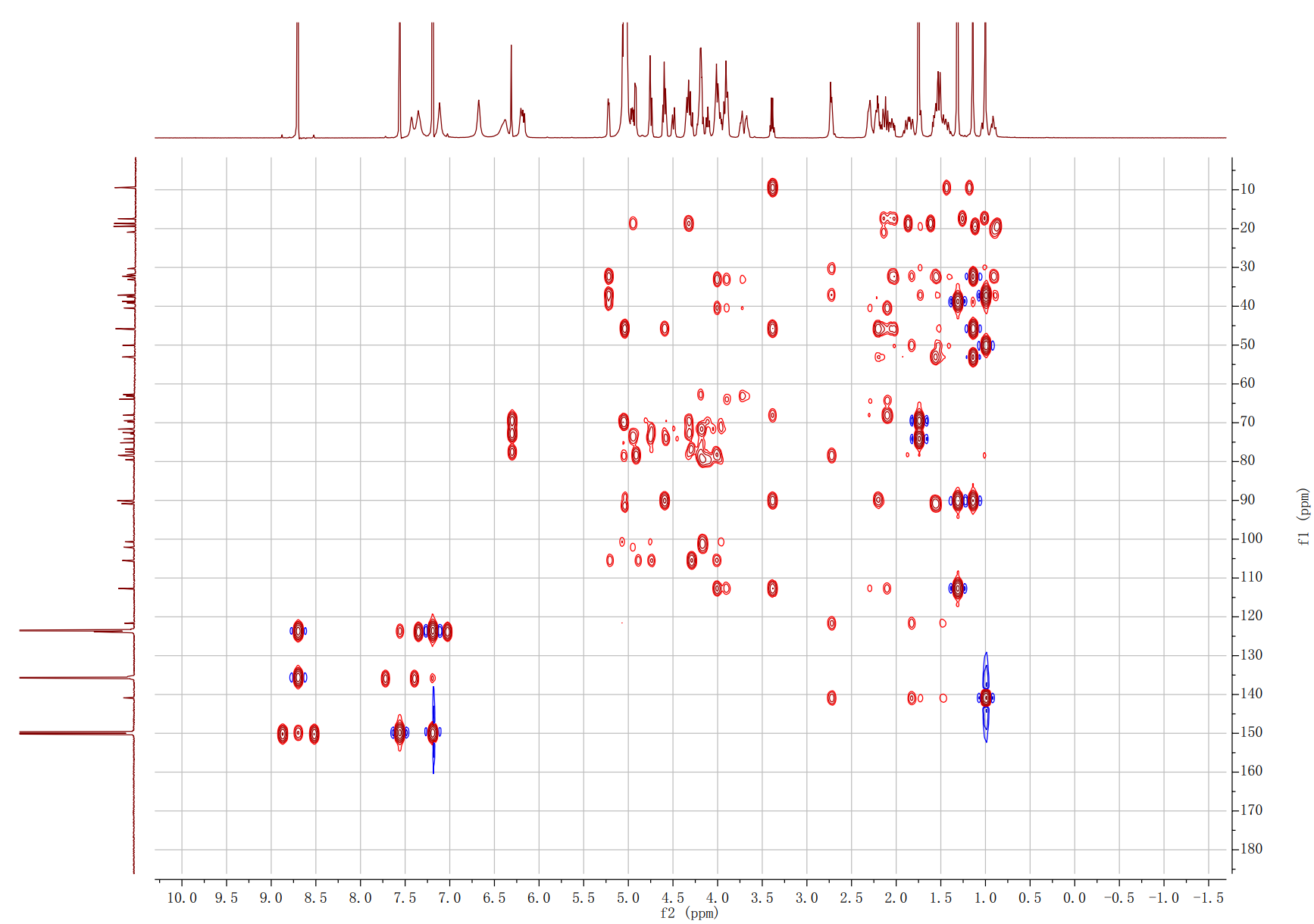


# **Fig. S19** HMBC spectrum of compound **3** in pyridine-*d*_5_.


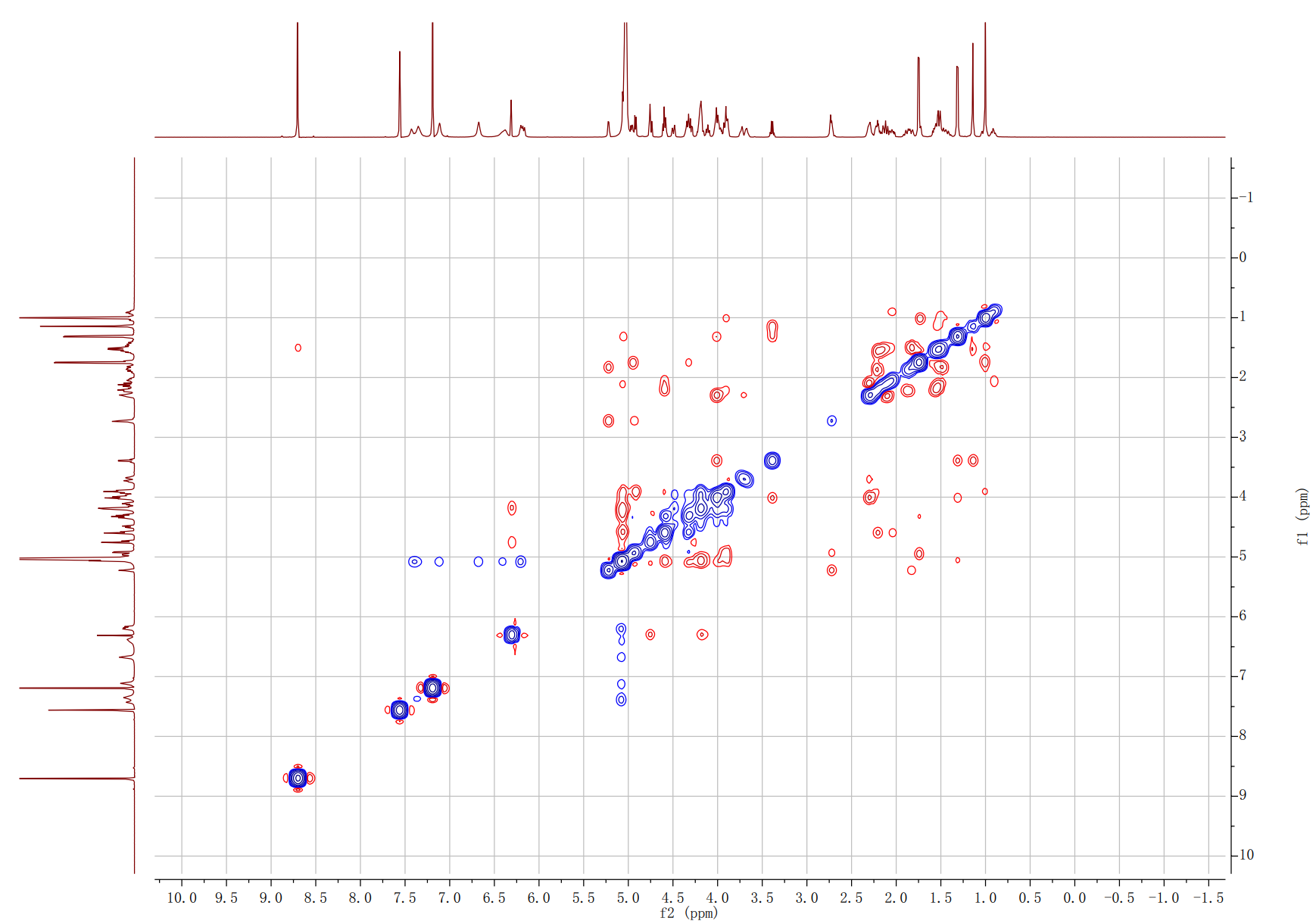


# **Fig. S20** ROESY spectrum of compound **3** in pyridine-*d*_5_.

_
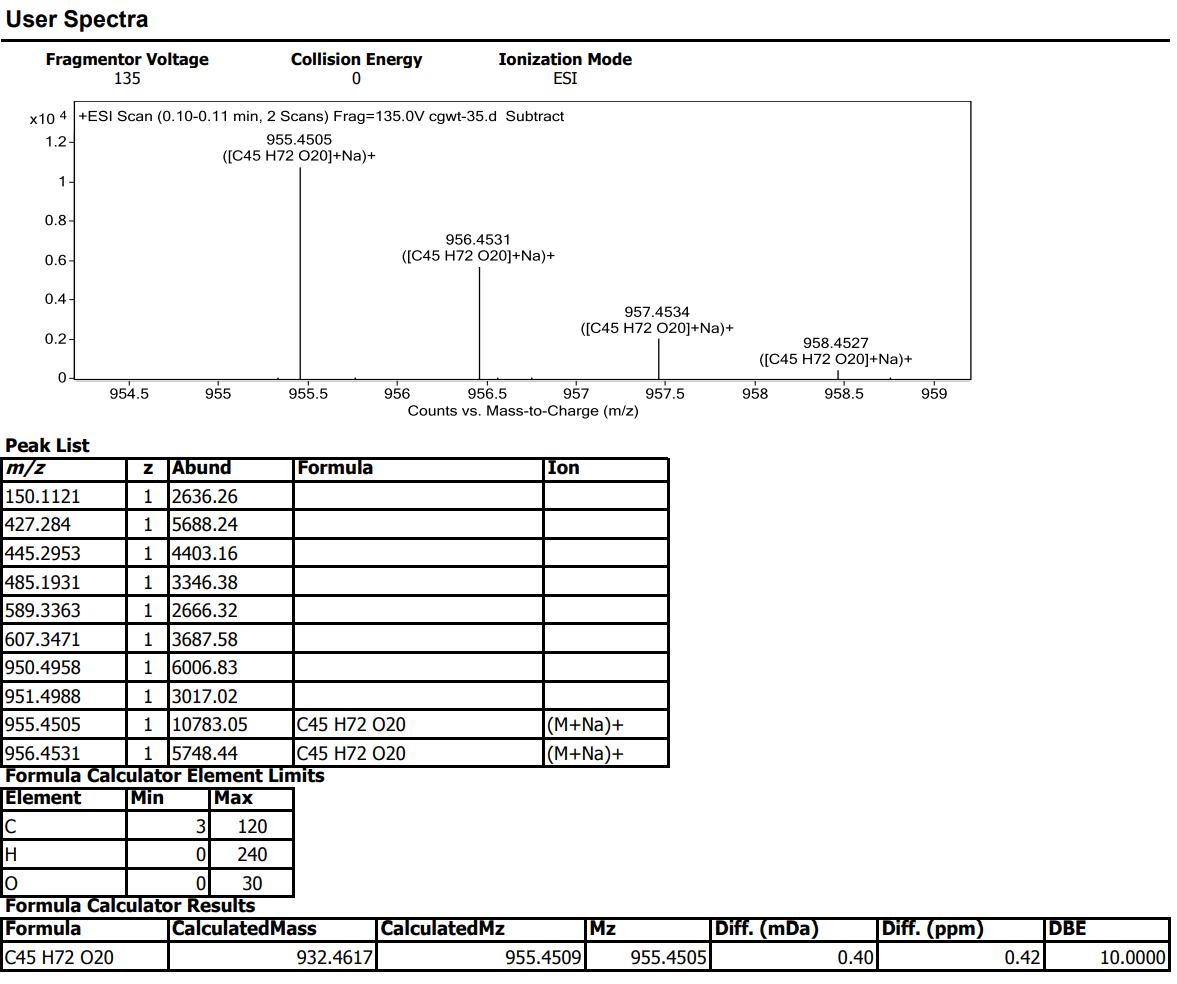
_

**Fig. S21** HRESI (+) MS spectrum of compound **3**.


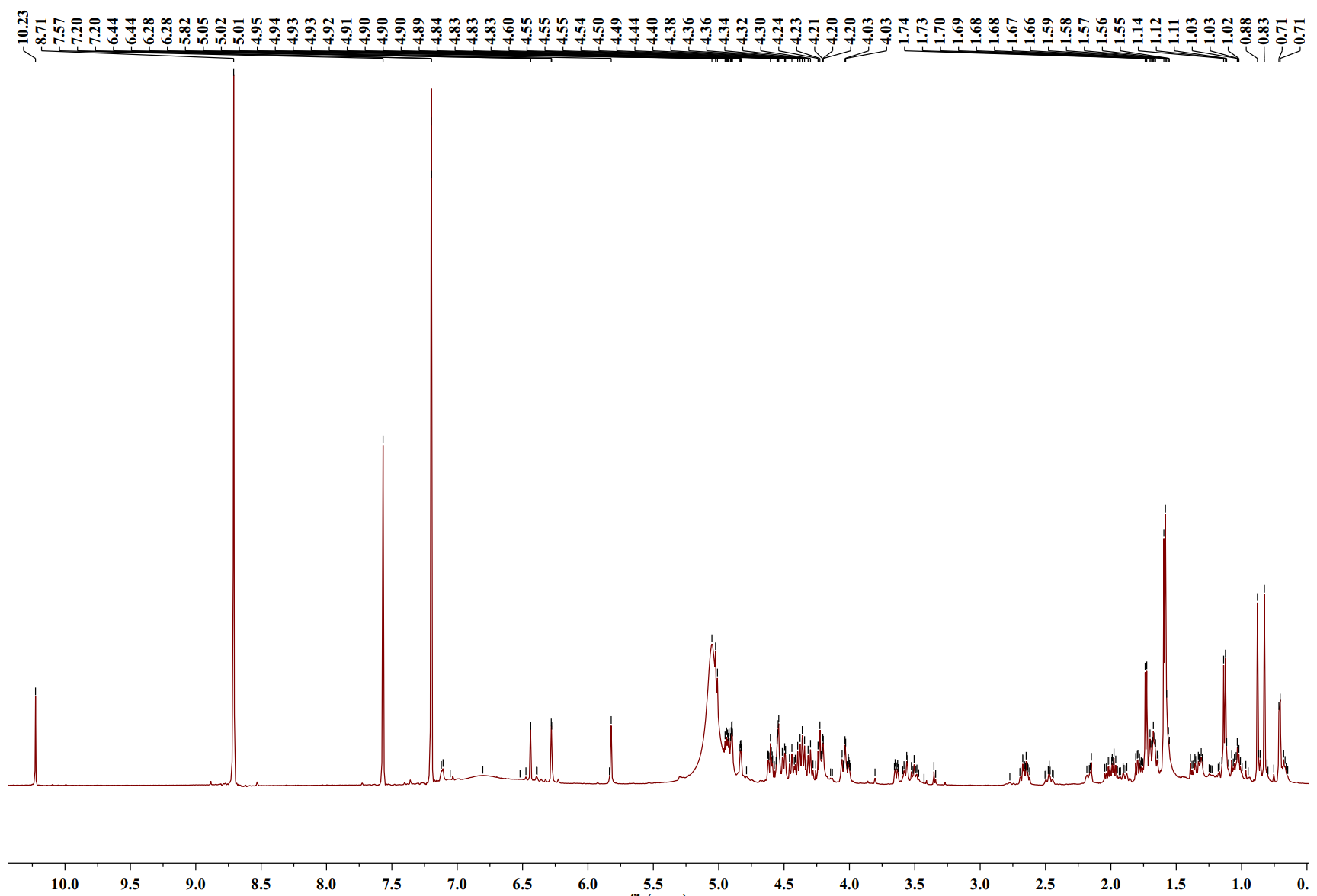


# **Fig. S22** ^1^H NMR spectrum (500 MHz) of compound **4** in pyridine-*d*_5_.


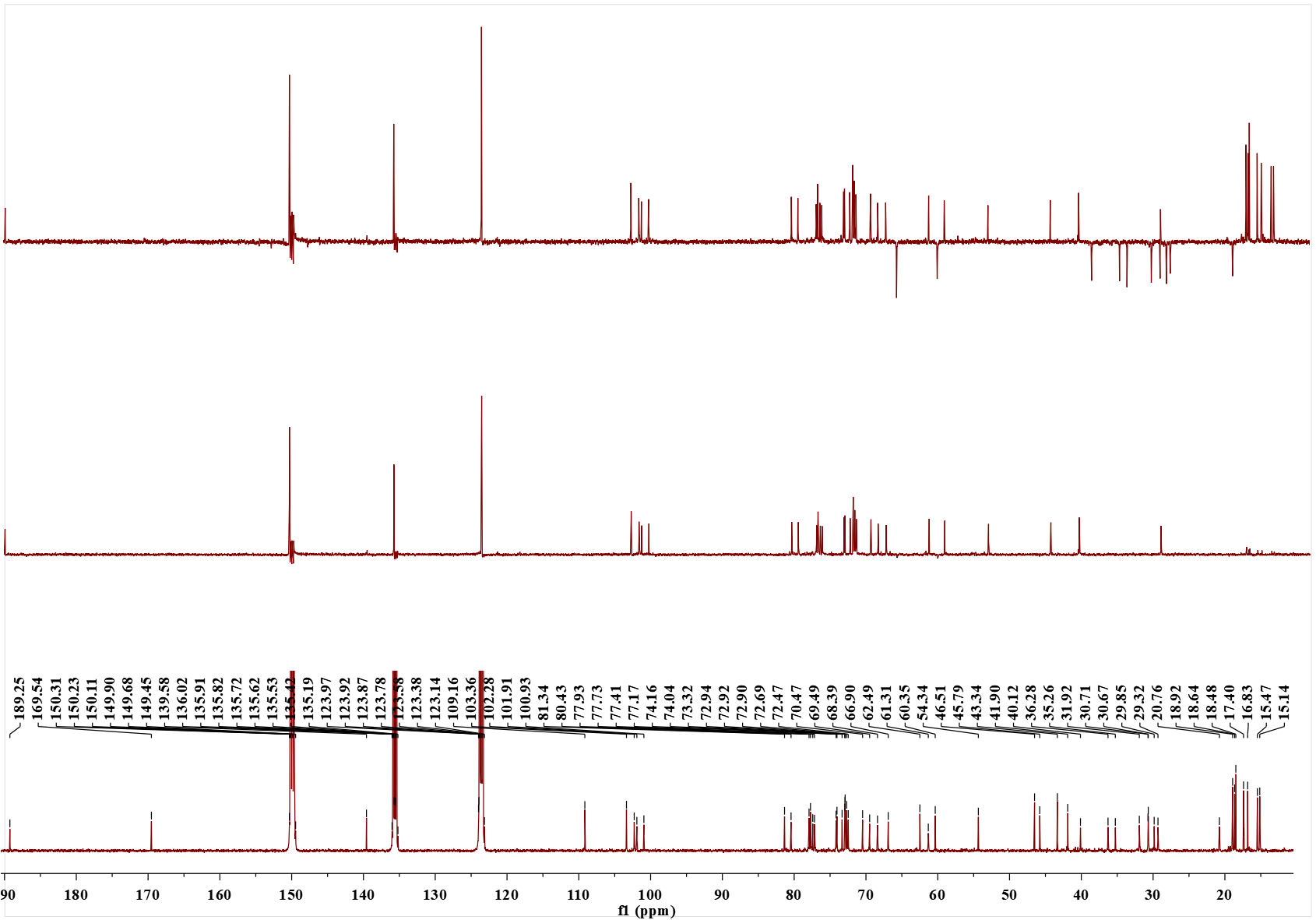


# **Fig. S23** ^13^C NMR spectrum (125 MHz) of compound **4** in pyridine-*d*_5_.


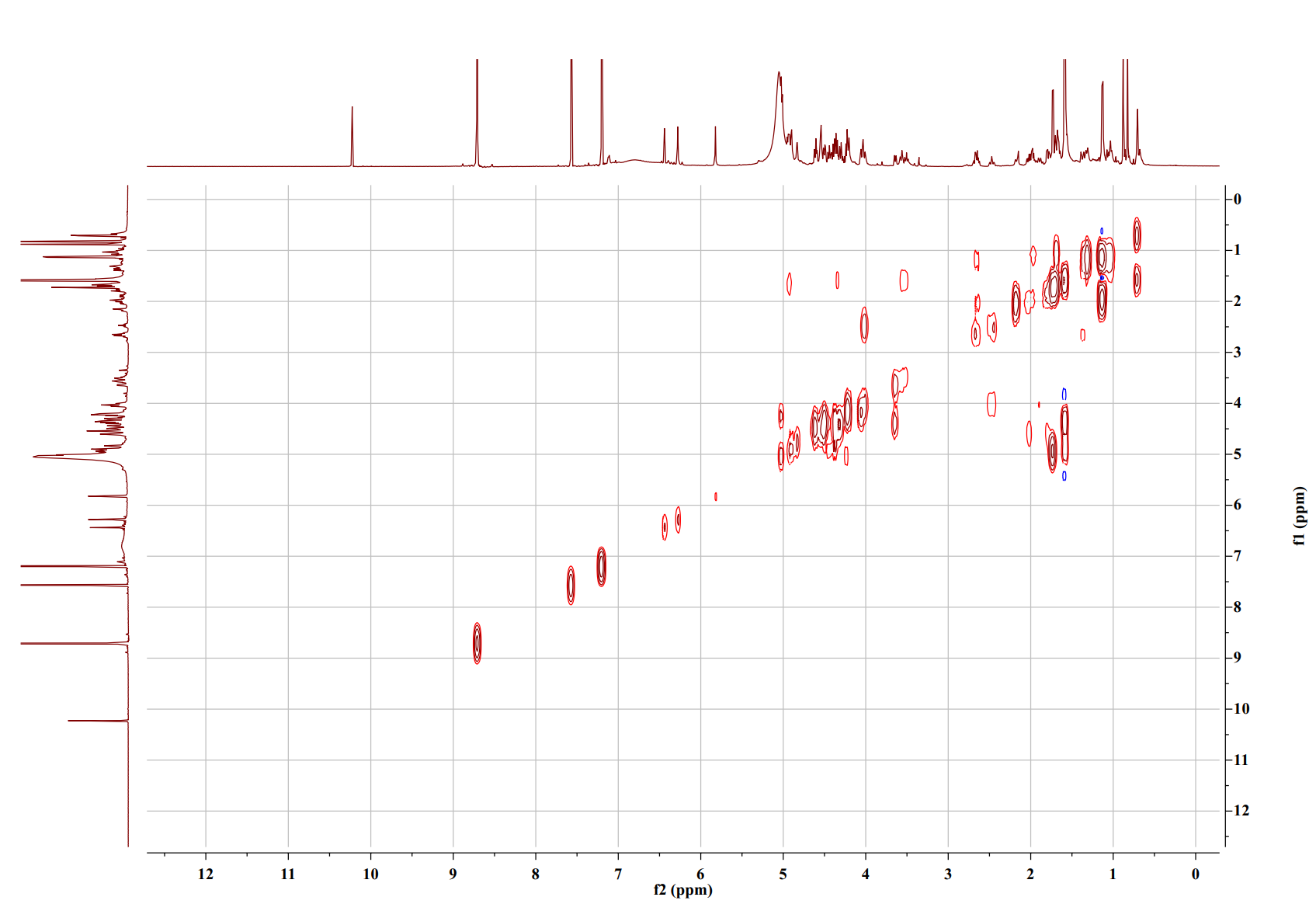


# **Fig. S24** ^1^H–^1^H COSY spectrum of compound **4** in pyridine-*d*_5_.


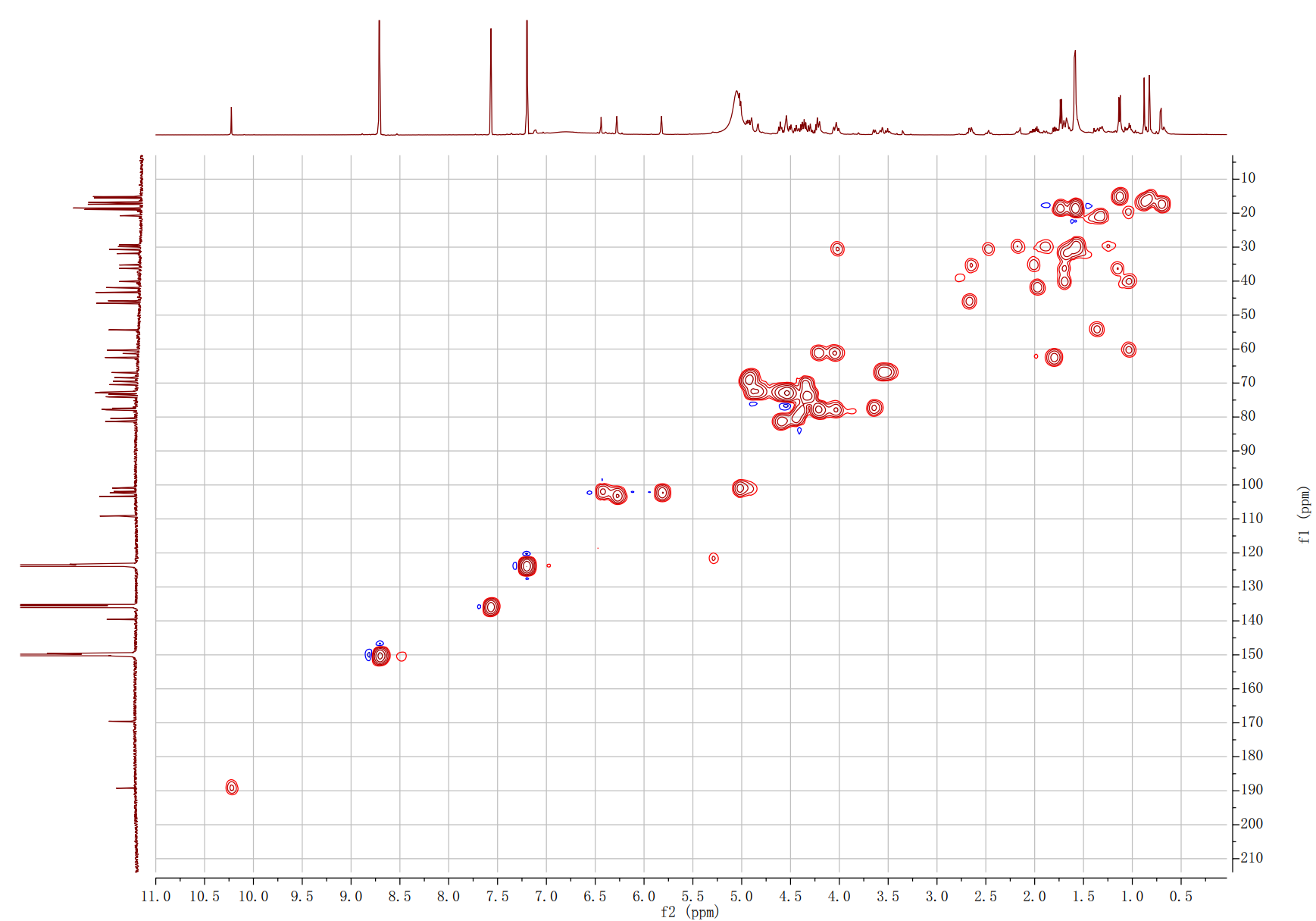


# **Fig. S25** HSQC spectrum of compound **4** in pyridine-*d*_5_.


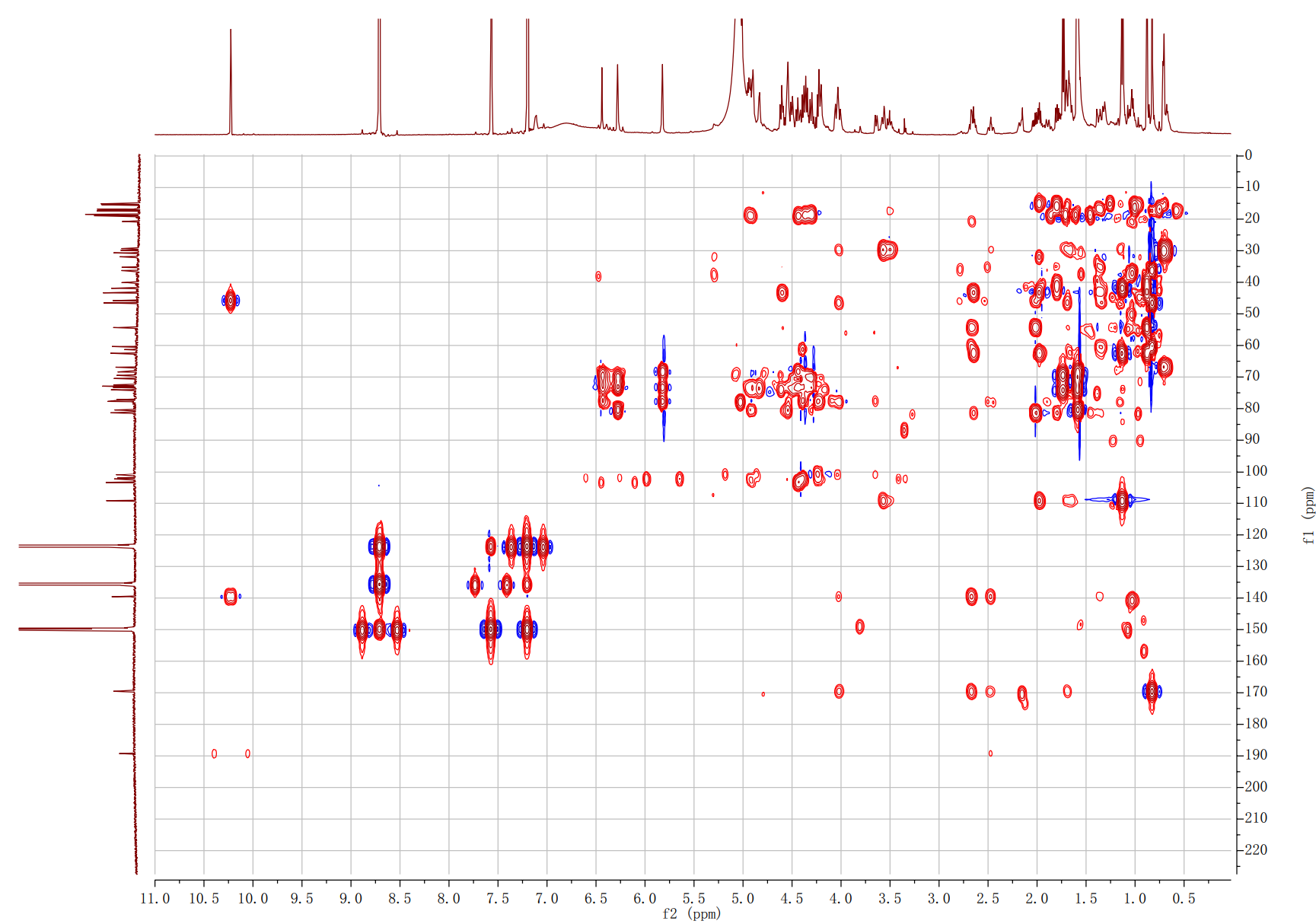


# **Fig. S26** HMBC spectrum of compound **4** in pyridine-*d*_5_.


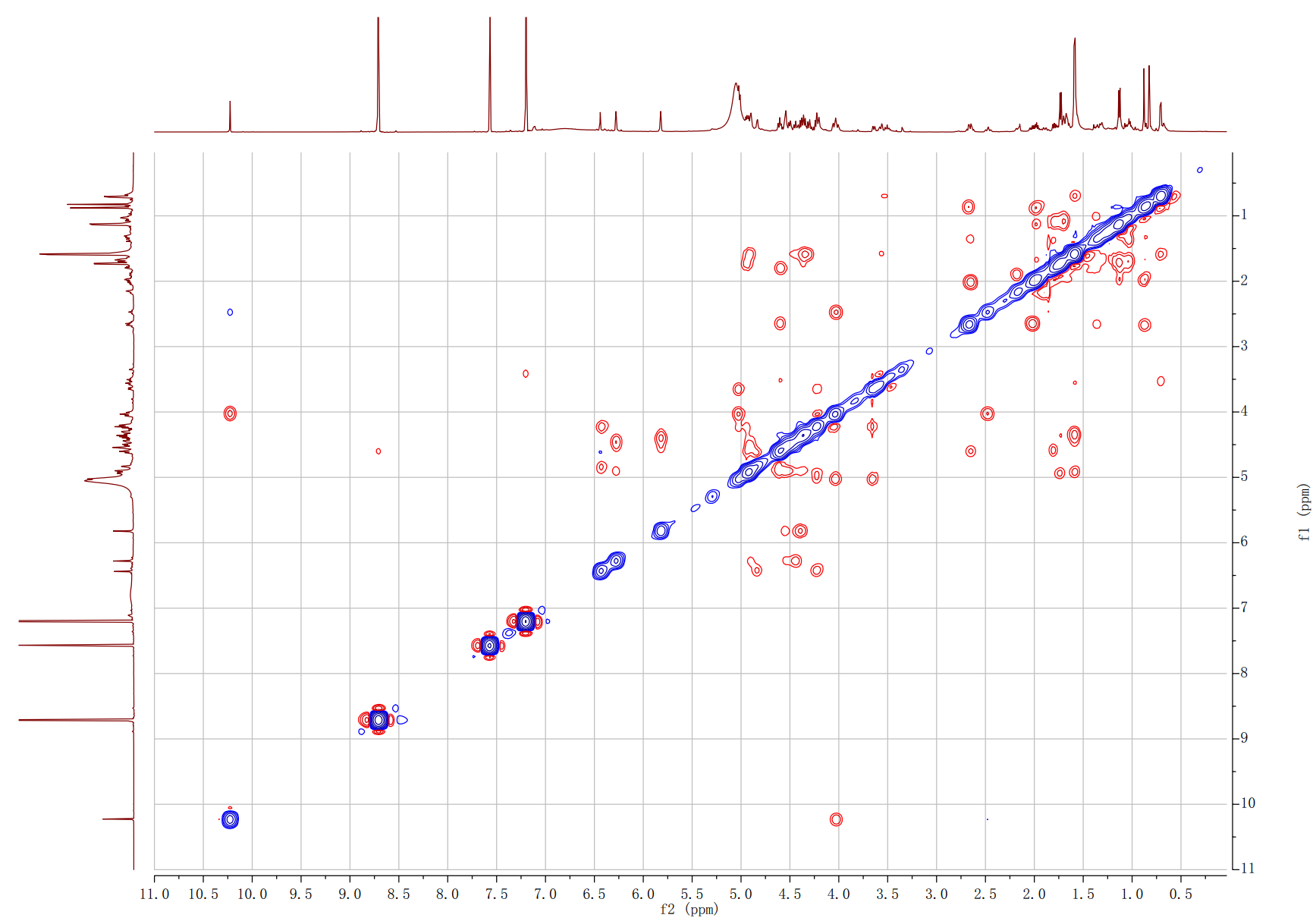


# **Fig. S27** ROESY spectrum of compound **4** in pyridine-*d*_5_.

_
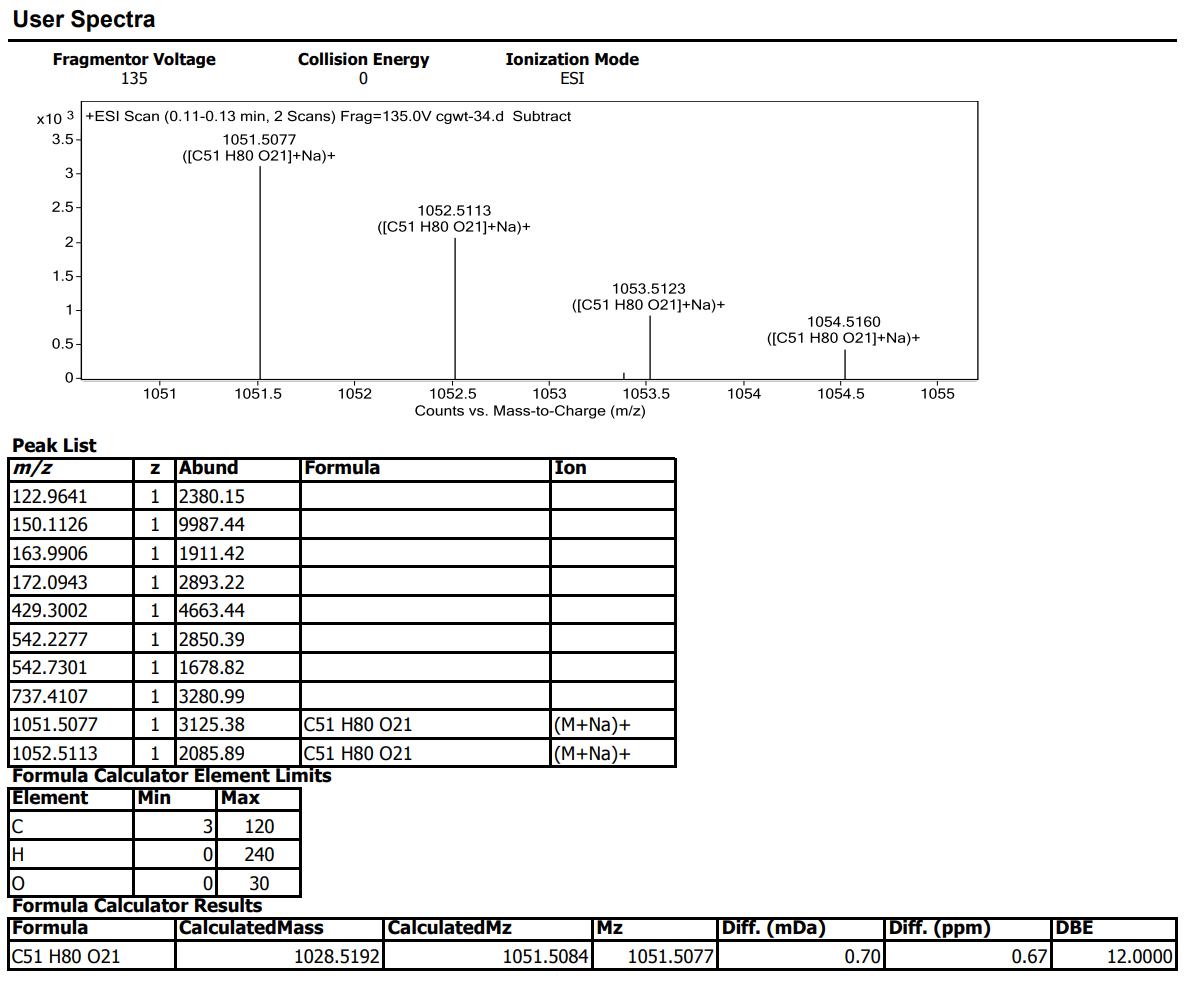
_

# **Fig. S28** HRESI (+) MS spectrum of compound **4**.


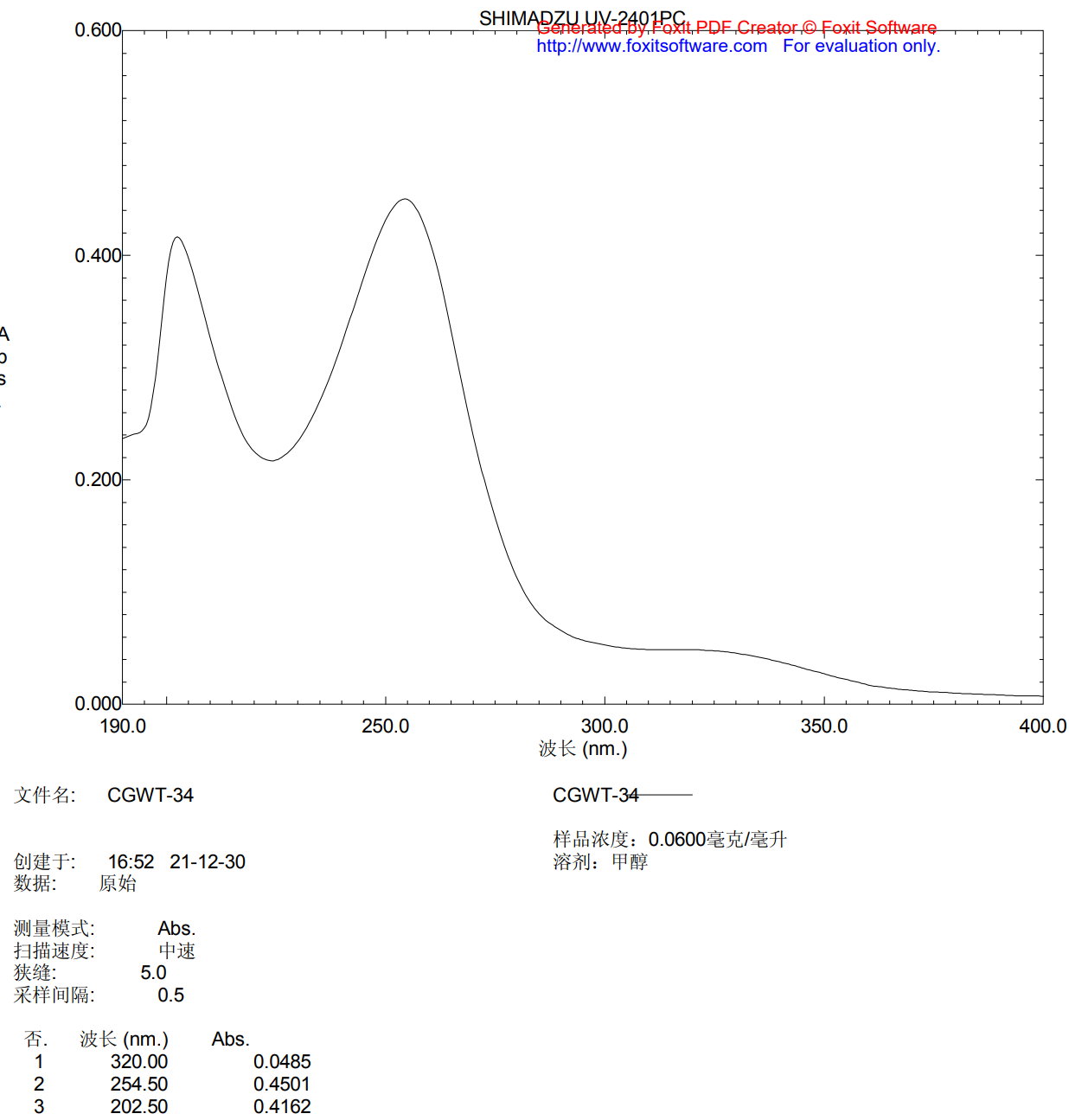


# **Fig. S29** UV spectrum of compound **4**.

**
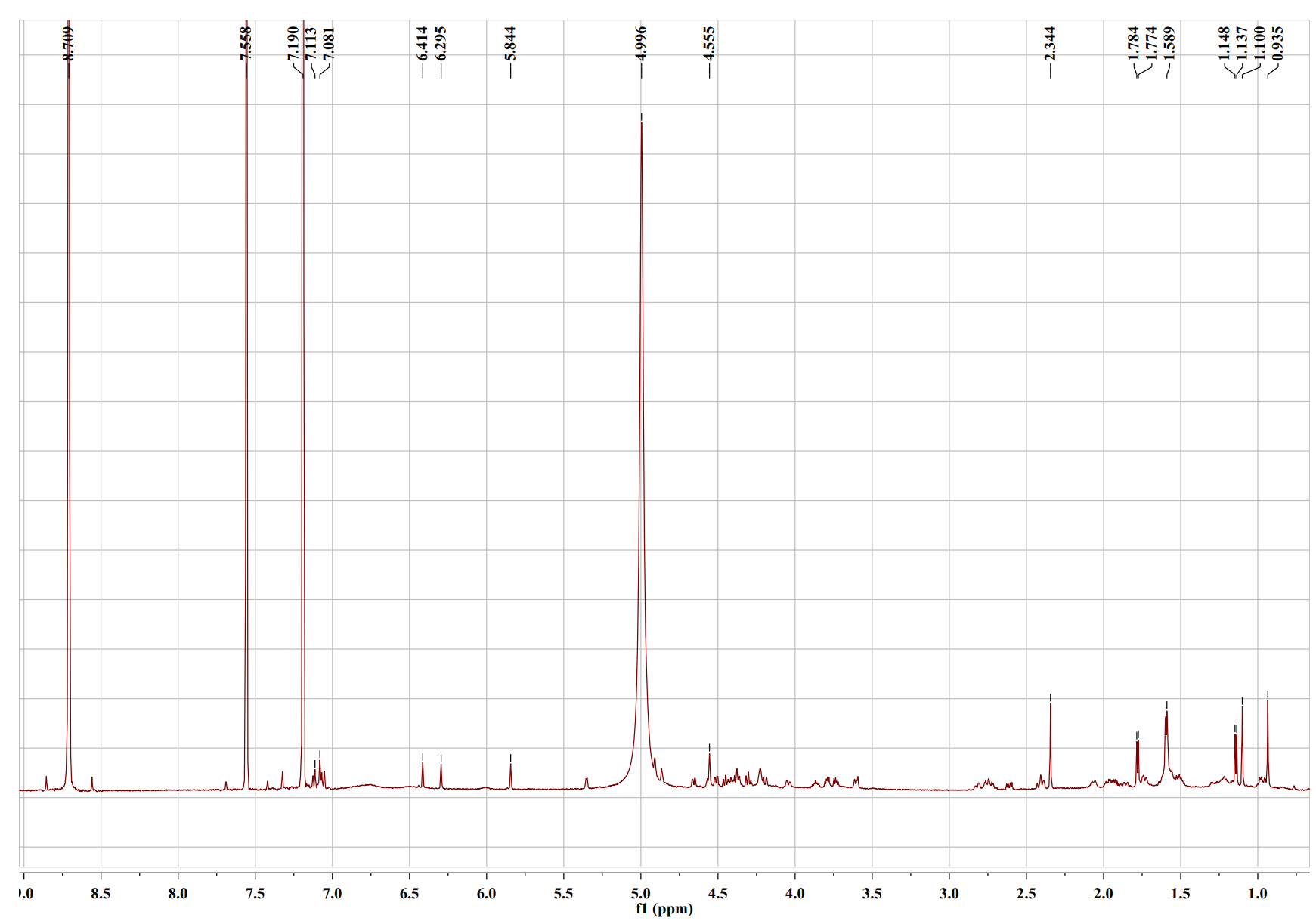
**

**Fig. S30** ^1^H NMR spectrum (600 MHz) of compound **5** in pyridine-*d*_5_.

**
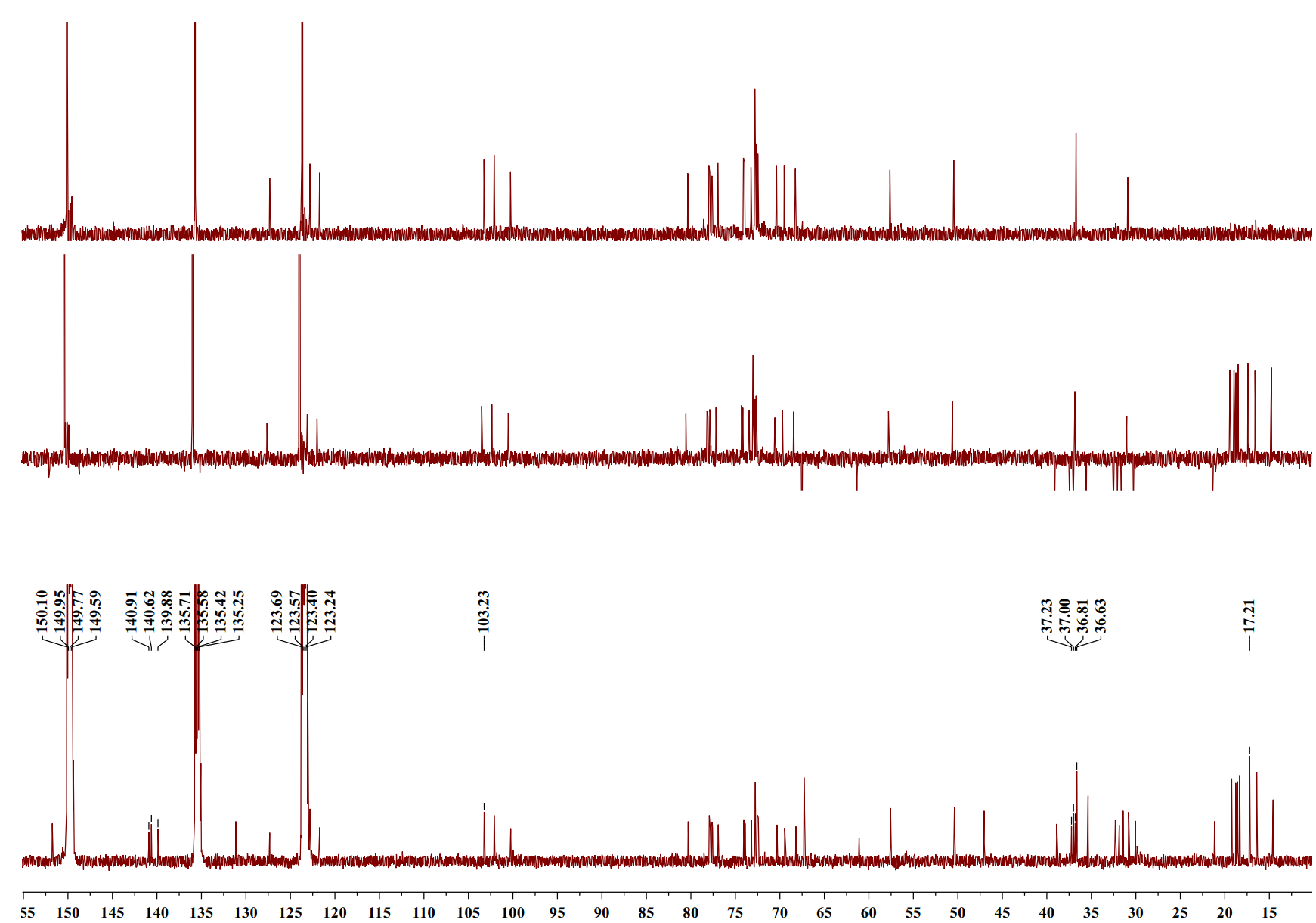
**

**Fig. S31** ^13^C NMR spectrum (150 MHz) of compound **5** in pyridine-*d*_5_.

**
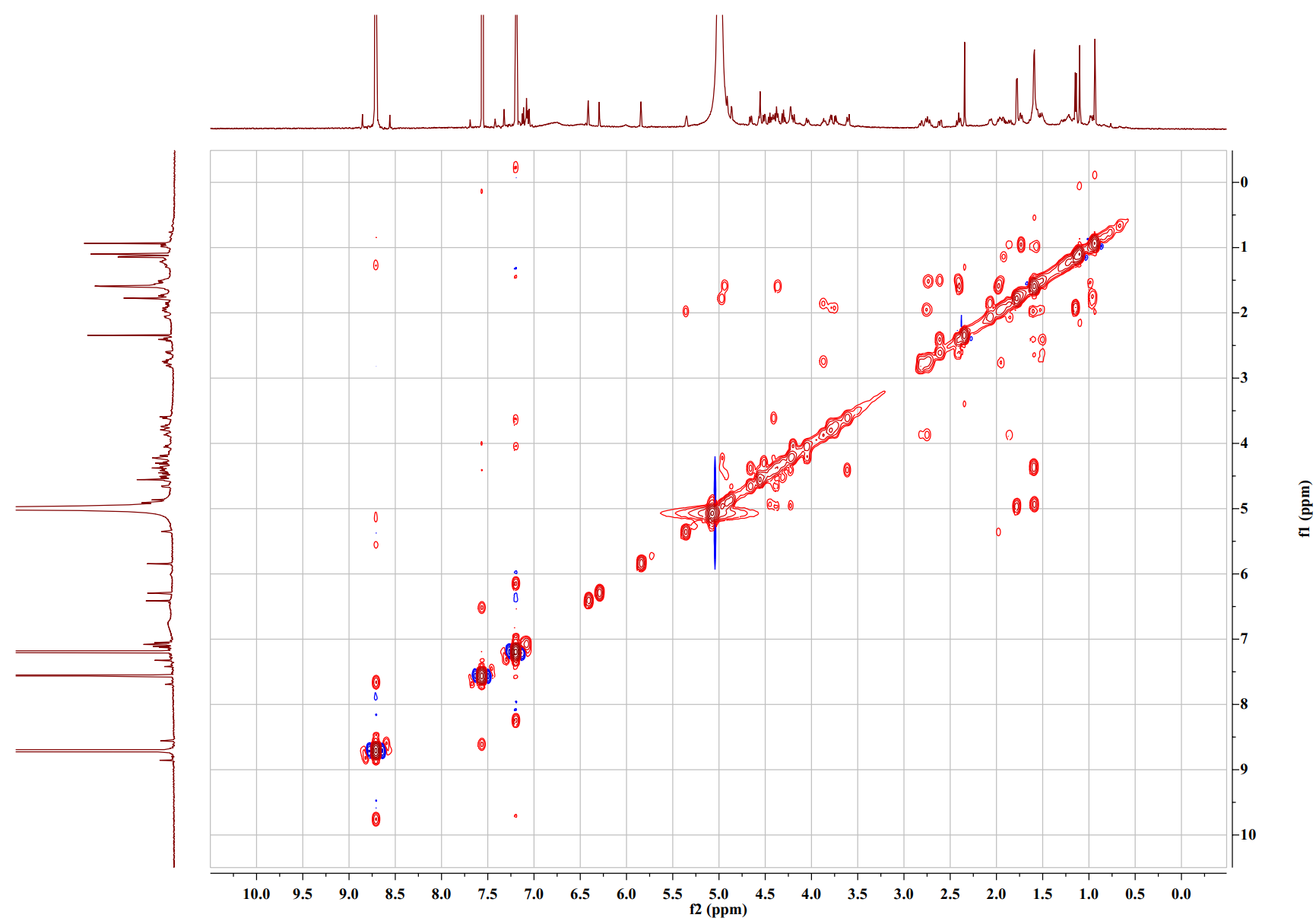
**

**Fig. S32** ^1^H–^1^H COSY spectrum of compound **5** in pyridine-*d*_5_.

**
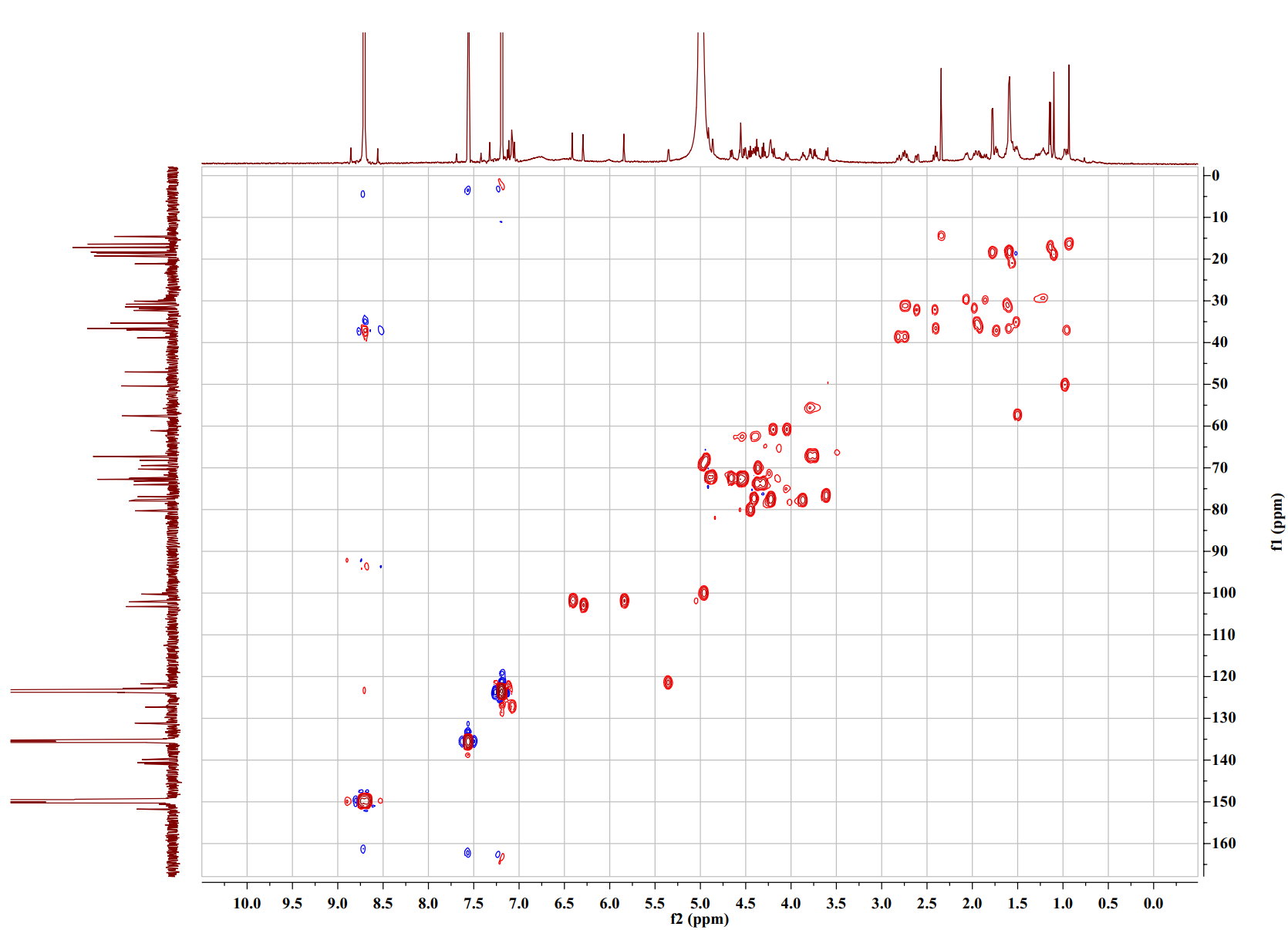
**

**Fig. S33** HSQC spectrum of compound **5** in pyridine-*d*_5_.

**
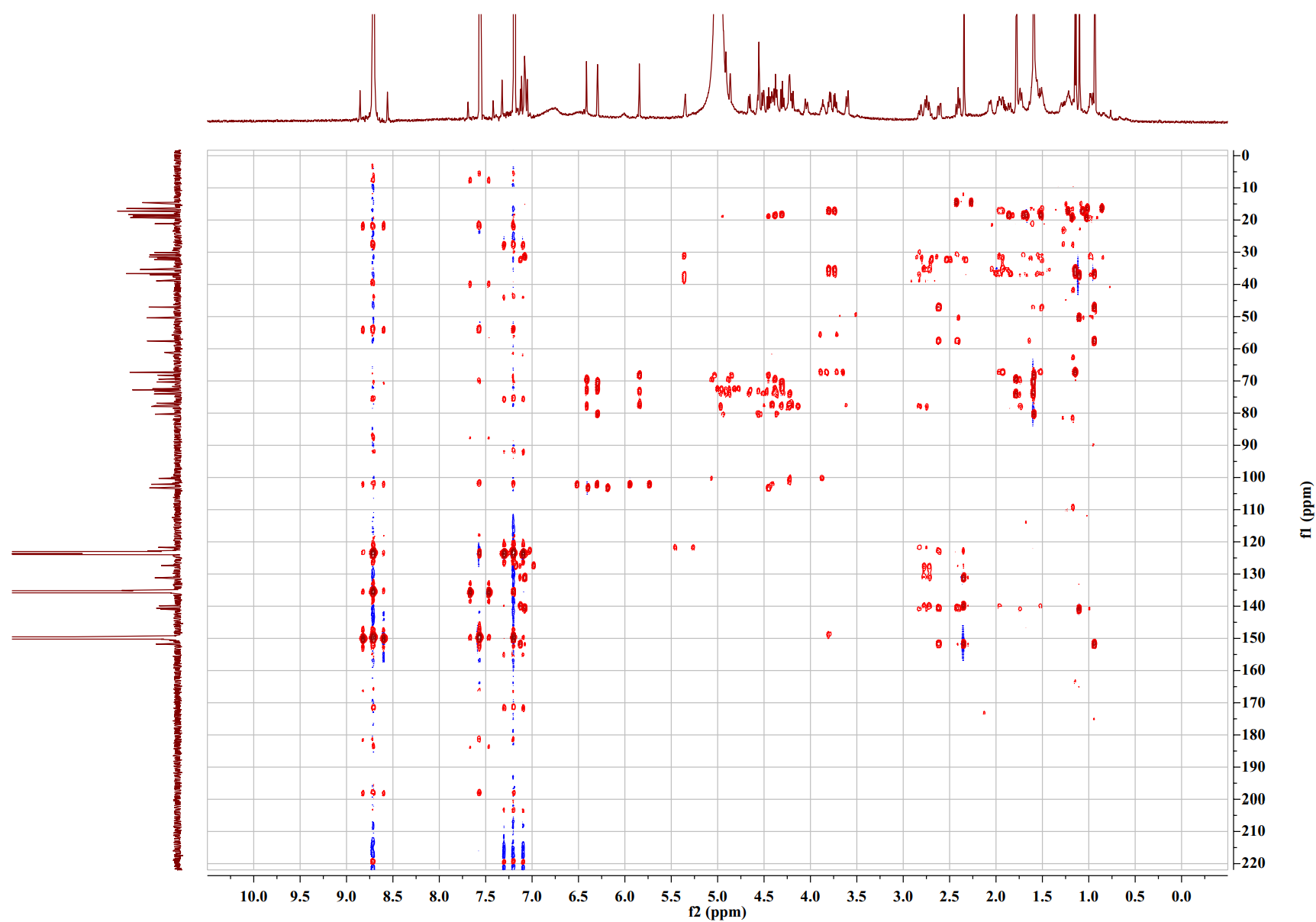
**

**Fig. S34** HMBC spectrum of compound **5** in pyridine-*d*_5_.

**
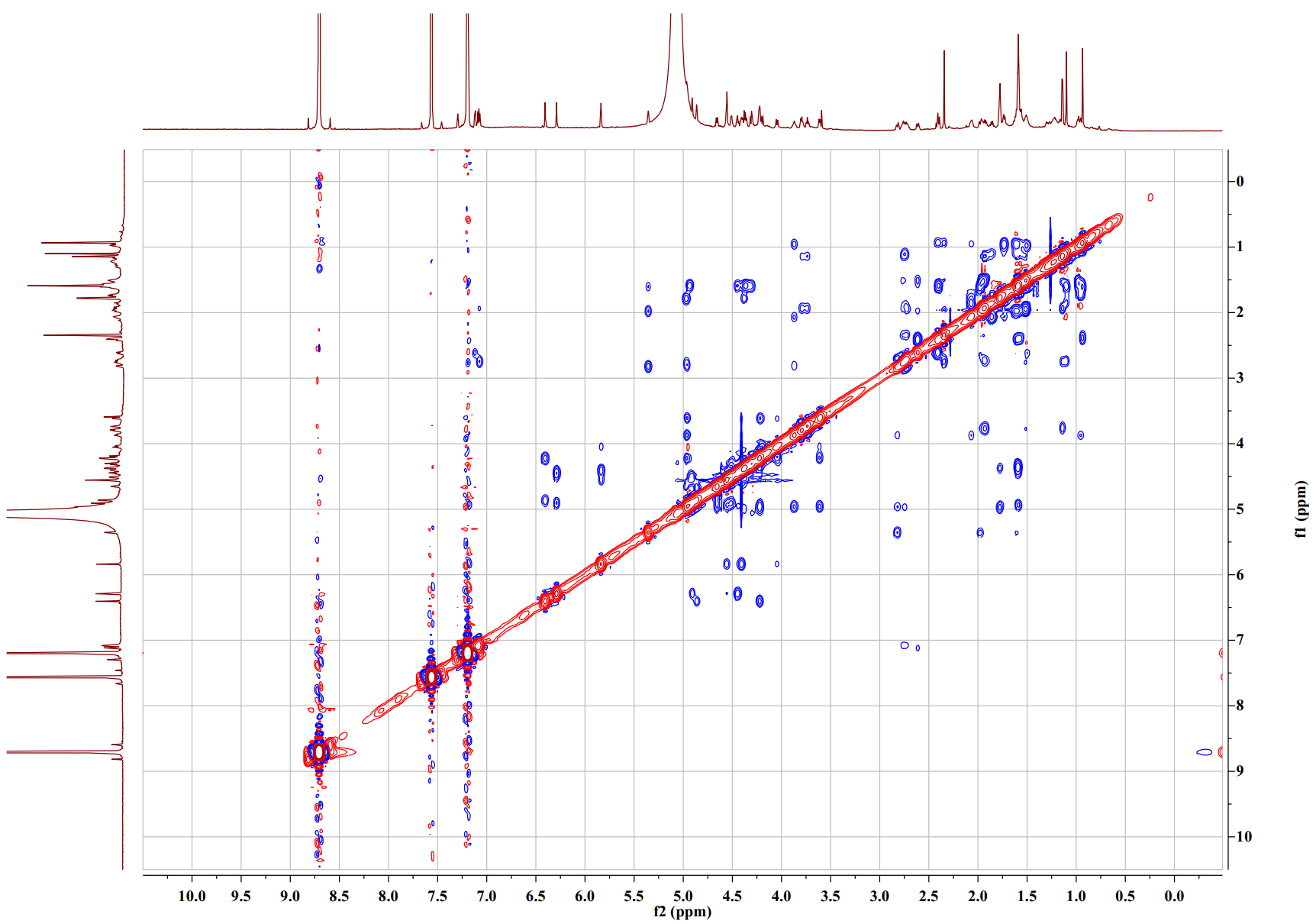
**

**Fig. S35** ROESY spectrum of compound **5** in pyridine-*d*_5_.

**_
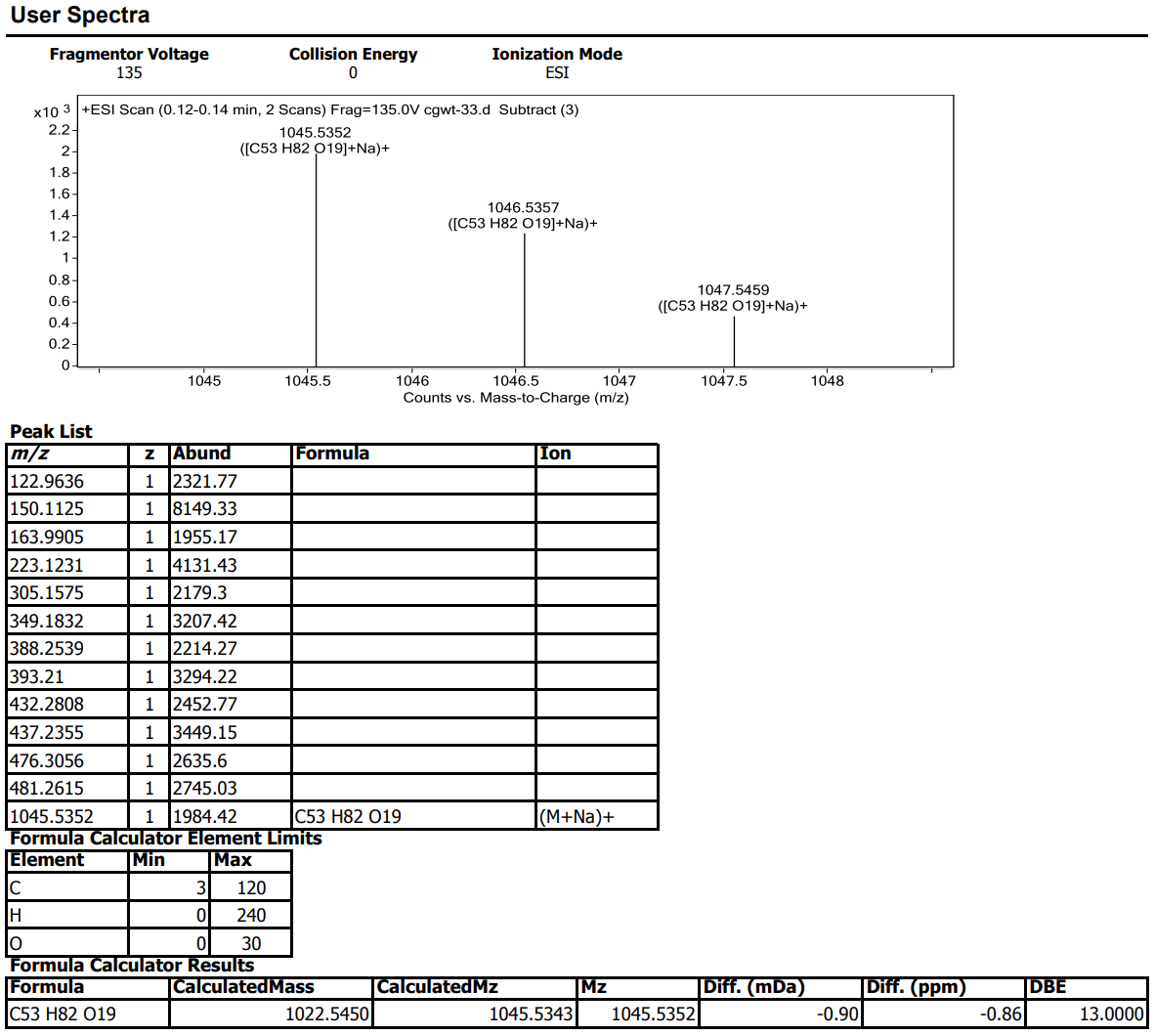
_**

**Fig. S36** HRESI (+) MS spectrum of compound **5**.

**
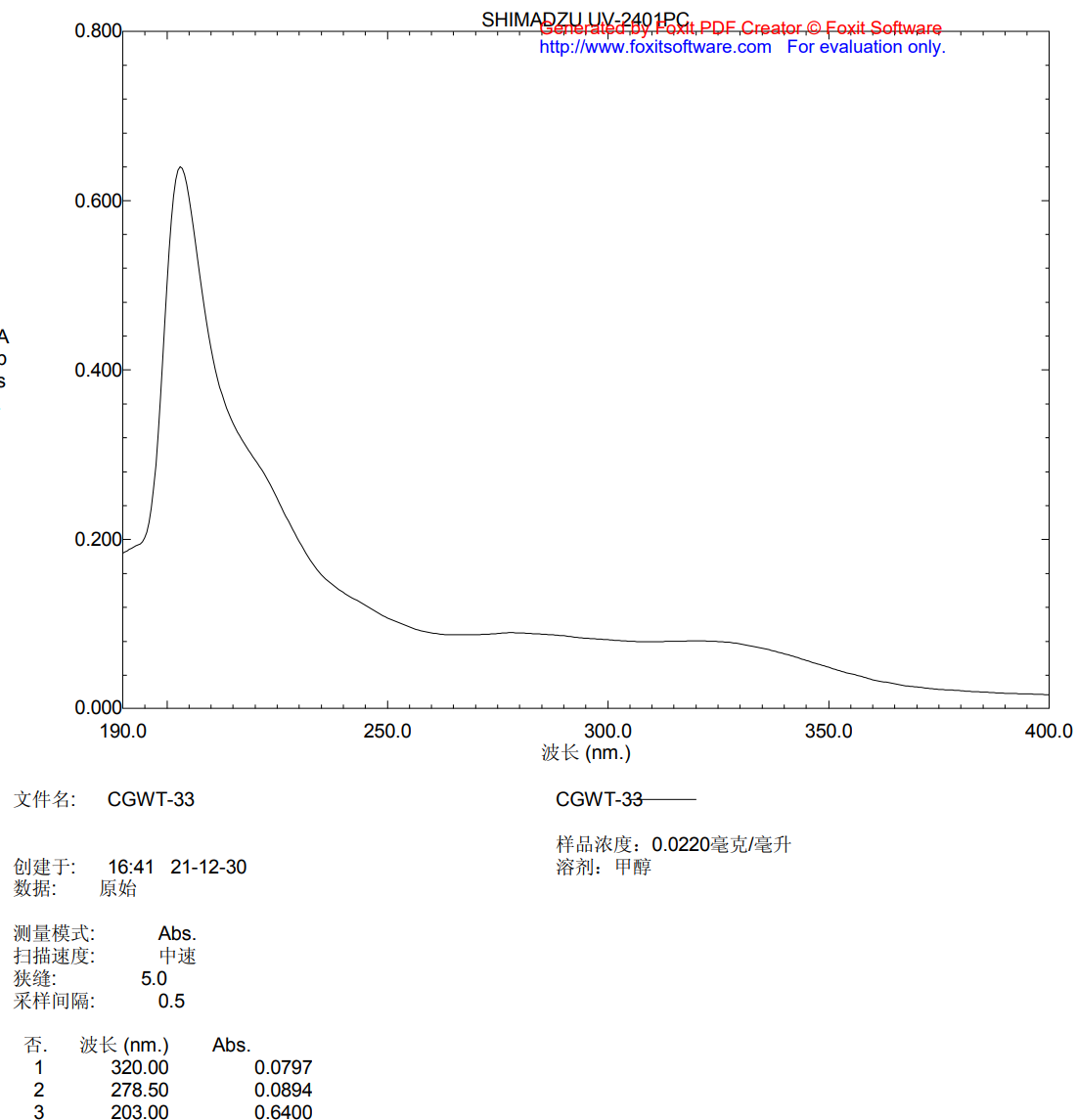
**

**Fig. S37** UV spectrum of compound **5**.
